# Molecular Evolution and Expression Divergence of the Aconitase (ACO) Gene Family in Land Plants

**DOI:** 10.3389/fpls.2016.01879

**Published:** 2016-12-12

**Authors:** Yi-Ming Wang, Qi Yang, Yan-Jing Liu, Hai-Ling Yang

**Affiliations:** ^1^Department of Biochemistry and Molecular Biology, College of Biological Sciences and Biotechnology, Beijing Forestry UniversityBeijing, China; ^2^State Key Laboratory of Systematic and Evolutionary Botany, Institute of Botany, Chinese Academy of SciencesBeijing, China

**Keywords:** aconitase, gene family, evolution, land plants, gene expression

## Abstract

Aconitase (ACO) is a key enzyme that catalyzes the isomerization of citrate to isocitrate in the tricarboxylic acid (TCA) and glyoxylate cycles. The function of ACOs has been well studied in model plants, such as *Arabidopsis*. In contrast, the evolutionary patterns of the ACO family in land plants are poorly understood. In this study, we systematically examined the molecular evolution and expression divergence of the ACO gene family in 12 land plant species. Thirty-six ACO genes were identified from the 12 land plant species representing the four major land plant lineages: Bryophytes, lycophytes, gymnosperms, and angiosperms. All of these ACOs belong to the cytosolic isoform. Three gene duplication events contributed to the expansion of the ACO family in angiosperms. The ancestor of angiosperms may have contained only one ACO gene. One gene duplication event split angiosperm ACOs into two distinct clades. Two clades showed a divergence in selective pressure and gene expression patterns. The *cis*-acting elements that function in light responsiveness were most abundant in the promoter region of the ACO genes, indicating that plant ACO genes might participate in light regulatory pathways. Our findings provide comprehensive insights into the ACO gene family in land plants.

## Introduction

The tricarboxylic acid (TCA) cycle is also called the Krebs cycle or the citric acid cycle. As in all eukaryotes, the TCA cycle in plants generates ATP, which is fundamental to supplying energy for organisms (Schnarrenberger and Martin, 2002). The TCA cycle is the central element of carbon metabolism, which provides electrons for oxidative phosphorylation and intermediates for amino acid biosynthesis. The TCA cycle also links the pathway of glycolysis to fatty acid metabolism via the glyoxylate cycle (Nunes-Nesi et al., 2013).

As the second step in the TCA cycle, the reversible isomerization of citrate to isocitrate via cis-aconitate as an intermediate is catalyzed by aconitase (Peyret et al., 1995). Aconitase (ACO, EC 4.2.1.3), also known as aconitate hydratase, is an iron-sulfur protein containing a 4Fe-4S cluster with typical molecular masses around 90 kDa (Beinert et al., 1996). Aconitase has two isoforms: Cytosolic aconitase and mitochondrial isoform. Mammalian aconitases have been well-studied, providing insight into the functions and mechanisms of this family (Matasova and Popova, 2008; Lushchak et al., 2014). In humans, ACO1 is a cytosolic aconitase, also called iron-responsive element-binding protein 1 (IRP1), which contains an AcnA_IRP domain. IRP1 can recognize the RNA stem-loop structure of an iron-responsive element (IRE), which is located at the 5′ un-translated region of ferritin mRNA, and inhibits translation of the ferritin transcript (Hentze and Kuhn, 1996). Thus, cytosolic aconitases are bifunctional proteins in animals; the aconitases show ACO activity when they assemble the 4Fe-4S cluster and switch to IRP activity when they disassemble the 4Fe-4S cluster. Additionally, in humans, ACO2 is a mitochondrial aconitase that functions in the Krebs cycle and contains an AcnA_Mitochondrial domain.

Notably, all aconitase sequences identified to date in higher plants are more similar to the cytosolic aconitase isoforms than to the mitochondrial isoforms in animals (Navarre et al., 2000; Arnaud et al., 2007; Moeder et al., 2007). All three aconitase proteins in *Arabidopsis* have the same AcnA_IRP domain with cytosolic aconitase IRP1 as humans; however no IRP activity was detected in the Arabidopsis aconitases (Arnaud et al., 2007). Three ACOs of *Arabidopsis* were detected in the mitochondrial proteome (Kruft et al., 2001; Heazlewood et al., 2004). Additionally, Aco-1 proteins of wild species of tomato were located in both the cytosol and mitochondria (Carrari et al., 2003). Because mitochondrial isoforms were not detected in *Arabidopsis*, tobacco, and tomato, the cytosolic aconitase with dual subcellular location may compensate for the aconitase activity in the mitochondria, as the mitochondrial isoforms do in animals (Carrari et al., 2003; Arnaud et al., 2007; Moeder et al., 2007).

In addition to the aconitase activity in the TCA cycle, plant aconitases participated in the carbon skeleton flow of lipid acid metabolism, sucrose metabolism, and the glyoxylate cycle (Hayashi et al., 1995; Eastmond and Graham, 2001; Borek and Nuc, 2011). Plant aconitases are also involved in several important physiological and developmental processes, including mediating oxidative stress and cell death, as they are the major intra-mitochondrial targets for inactivation by H_2_O_2_, a key NO sensor that activates the hypersensitive response (HR) (Verniquet et al., 1991; Navarre et al., 2000; Arnaud et al., 2007; Moeder et al., 2007).

Because ACOs had been studied in few model plants and only cytosolic ACOs were observed in higher plants, the evolutionary patterns of the ACO family in land plants are poorly understood (Arnaud et al., 2007; Terol et al., 2010). With the availability of various plant genome data, an integrated genome-wide phylogenetic analysis of ACO genes from bryophytes (*Physcomitrella patens*), lycophytes (*Selaginella moellendorffii*), gymnosperms, and angiosperms will help us to understand the evolutionary history and functional divergence of this gene family in land plants. Three gymnosperm species were chosen for this study: *Picea abies, Picea glauca*, and *Pinus taeda*. Additionally, seven angiosperm species were selected, including four monocots (*Sorghum bicolor, Zea mays, Oryza sativa*, and *Brachypodium distachyon*) and three eudicots (*Arabidopsis thaliana, Populus trichocarpa*, and *Glycine max*). We conducted a genome-wide study of ACO genes in the 12 selected land plant species. The molecular evolution and functional divergence of the ACO family were investigated by integrating gene sequences, phylogenetic analysis, molecular evolution, gene expression patterns, and *cis*-acting element analysis in the different land plant species. Our study sheds light on the evolution of ACOs in land plants and provides a useful framework for further functional characterization.

## Materials and methods

### ACO gene identification and characterization

To identify the ACO gene family members in land plants, three ACO protein sequences of *A. thaliana* (AT4G35830, AT4G26970, and AT2G05710) were used for a TBLASTN search using the default algorithm parameters. The TBLASTN search was performed for eight land plant species (*P. patens, S. moellendorffii, B. distachyon, O. sativa, S. bicolor, Z. mays, P. trichocarpa*, and *G. max*) using the Phytozome v11.0 database (Goodstein et al., 2012) and for three gymnosperm species (*P. abies, P. glauca*, and *P. taeda*) using the ConGenIE database (Nystedt et al., 2013; Sundell et al., 2015). We also performed the same search for two green algae species (*Chlamydomonas reinhardtii* and *Volvox carteri*) using the Phytozome database to use as an outgroup in phylogenetic analysis. Additionally, we downloaded the multiple sequence alignment of the ACO conserved domain (PF00330) from Pfam (http://pfam.xfam.org). Subsequently, this sequence alignment was used to generate a Hidden Markov Model (HMM) profile using HMMER 3.1b2 (http://hmmer.org). This HMM profile was applied to identify ACO proteins via the hmmsearch program (HMMER 3.1b2, http://hmmer.org) against plant protein databases; these protein databases were downloaded from the Phytozome and the ConGenIE. All collected ACO candidates were checked and corrected by the Expressed Sequence Tag (EST) databases of the National Center for Biotechnology Information (NCBI). Then, all ACO candidates were analyzed using the conserved domain search in the NCBI (http://www.ncbi.nlm.nih.gov/Structure/cdd/wrpsb.cgi), SMART (http://smart.embl-heidelberg.de), and PROSITE (http://prosite.expasy.org) web servers to confirm the presence of aconitase domains in their protein structures.

To study the structures of individual ACO genes, the exon/intron organizations were obtained using the Gene Structure Display Server 2.0 (GSDS, http://gsds.cbi.pku.edu.cn). The exon/intron organization for ACO genes were drawn by comparing the cDNA sequences with their corresponding genomic DNA sequences.

### Phylogenetic and molecular evolution analyses

The ACO protein sequences were aligned (Supplemental Figure S1) using the MUSCLE program (Edgar, 2004) and manually adjusted using BioEdit v7.0.0 software (Hall, 1999). By using the protein sequence alignment to construct the corresponding coding sequence alignment, the multiple alignment of coding DNA was performed using the RevTrans 2.0 (http://www.cbs.dtu.dk/services/RevTrans-2.0/web, Wernersson and Pedersen, 2003). The maximum likelihood tree of aligned DNA sequences was constructed using PhyML v2.4.4 software (Guindon and Gascuel, 2003) with one thousand bootstrap replicates. We used the GTR model as the optimal DNA substitution model which was selected by modelgenerator version 0.85 (Keane et al., 2006). The two green algae ACO genes were used as an outgroup for phylogenetic analysis.

The ω values (ω = dN/dS) among all pairwise comparisons within each of the two domains (AcnA_IRP domain and AcnA_IRP_Swivel domain) were calculated using the YN00 program within the PAML 4.3 package (Yang, 2007). The calculations were performed separately using the AcnA_IRP domain (from alignment position 220 to alignment position 711 in Supplemental Figure S1), and the AcnA_IRP_Swivel domain (from alignment position 816 to alignment position 965 in Supplemental Figure S1). A two-sample *t*-test was performed to determine whether the ω values of the two domains were significantly different. To evaluate the selective pressures between the angiosperm clades A1 and A2, the branch models of CODEML in PAML were used to estimate ω values under two assumptions: A one-ratio model that assumes the same ω ratio for two clades, and a two-ratio model that assumes different ω ratios for the two clades. To verify which model best fit the data, likelihood ratio tests (LRTs) were performed by comparing twice the difference in the log-likelihood values between pairs of the models using a χ^2^ distribution (Yang and Nielsen, 2000).

### Expression of ACO genes

To investigate the expression patterns of the land plant ACO genes, we first examined the expression profiles of *P. patens, O. sativa, Z. mays, P. trichocarpa, A. thaliana*, and *G. max* in the eFP Browser (http://bar.utoronto.ca) and the expression profiles of *B. distachyon* and *S. bicolor* in the Phytozome v11.0 database (Goodstein et al., 2012). Based on a comparison of the relative expression values with the highest expression value in the corresponding gene expression data set, we then constructed heat maps for each of the eight species with the obtained gene expression data sets. To examine the tissue specific expression patterns of *S. moellendorffii*, total RNAs were isolated from root, stem, and leaf tissues of 2 year-old cultivated plants. The isolated RNAs were then treated with RNase-free DNase I (Promega) and reverse transcribed into cDNA using the RNA PCR Kit (AMV) version 3.0 (TaKaRa). Specific PCR primers were designed (Supplemental Table S1) based on the multiple sequence alignment of two *S. moellendorffii* ACO gene sequences. The actin gene (Accession NO. in NCBI: XM_002976779.1) was used as an internal control. A volume of 25 μL PCR mixture containing 3 μL first-strand cDNA, 2.5 μL TaKaRa 10 × PCR buffer, 0.125 μL TaKaRa Ex Taq (5 μL), 2 μL dNTP (2.5 mM each), and 0.4 pmol primer. PCR conditions were optimized to an initial denaturation of 3 min at 94°C, followed by 35 cycles of 30 s at 94°C, 40 s at 60°C and 1 min at 72°C with a final extension of 3 min at 72°C. The PCR products were analyzed using a 1% agarose gel and were validated by DNA sequencing. Independent biological triplicates were performed. To examine the expression of the ACO genes from three gymnosperms (*P. abies, P. glauca*, and *P. taeda*), the expression profiles of *P. abies* were obtained using the ConGenIE database (Sundell et al., 2015). And the NCBI EST expression data for *P. glauca* and *P. taeda* were used in the gene expression analysis.

### *Cis*-acting element analyses

The potential promoter region (from the start codon to 1500 bp upstream) of each ACO gene was obtained from Phytozome v11.0 (Goodstein et al., 2012); the only exception was the *PgACO1* gene, as we did not find the start codon of this gene from the *P. glauca* genome. The *cis*-acting elements of putative promoters were identified using the PlantCARE program (http://bioinformatics.psb.ugent.be/webtools/plantcare/html). We then analyzed the types and numbers of *cis*-acting elements of the gene promoter.

## Results

### Identification of the ACO gene family from land plants

In this study, 36 full-length genes encoding putative ACOs were identified from 12 land plant species (Supplemental Table S2). These land plant species represent the four major land plant lineages: Bryophytes, lycophytes, gymnosperms, and angiosperms. Among the 36 putative ACO genes, only one (*ZmACO1*) from *Z. mays* was considered to be a putative pseudogene, because of a frame shift disrupting the coding region and resulting in a truncated protein. After revising the frame shift by deleting one nucleotide, this gene was included in the phylogenetic analyses. Domain analysis performed by searching the SMART and PROSITE databases showed all 36 putative ACO genes encoded the aconitase domain, indicating that they belonged to the ACO gene family. The NCBI conserved domain search showed that all the predicted proteins encoded by the 36 genes contained AcnA_IRP and AcnA_IRP_Swivel domains. The AcnA_IRP and AcnA_IRP_Swivel domains were the same domains as in the cytosolic isoform of animals. We also identified ACO genes from the *C. reinhardtii* and *V. carteri* genomes; these two green algae ACO genes were used as outgroups in the phylogenetic analyses. Domain analysis showed that these two green algae ACOs contained the same AcnA_Mitochondrial domains as in the mitochondrial isoforms of animals.

**Figure 1:**
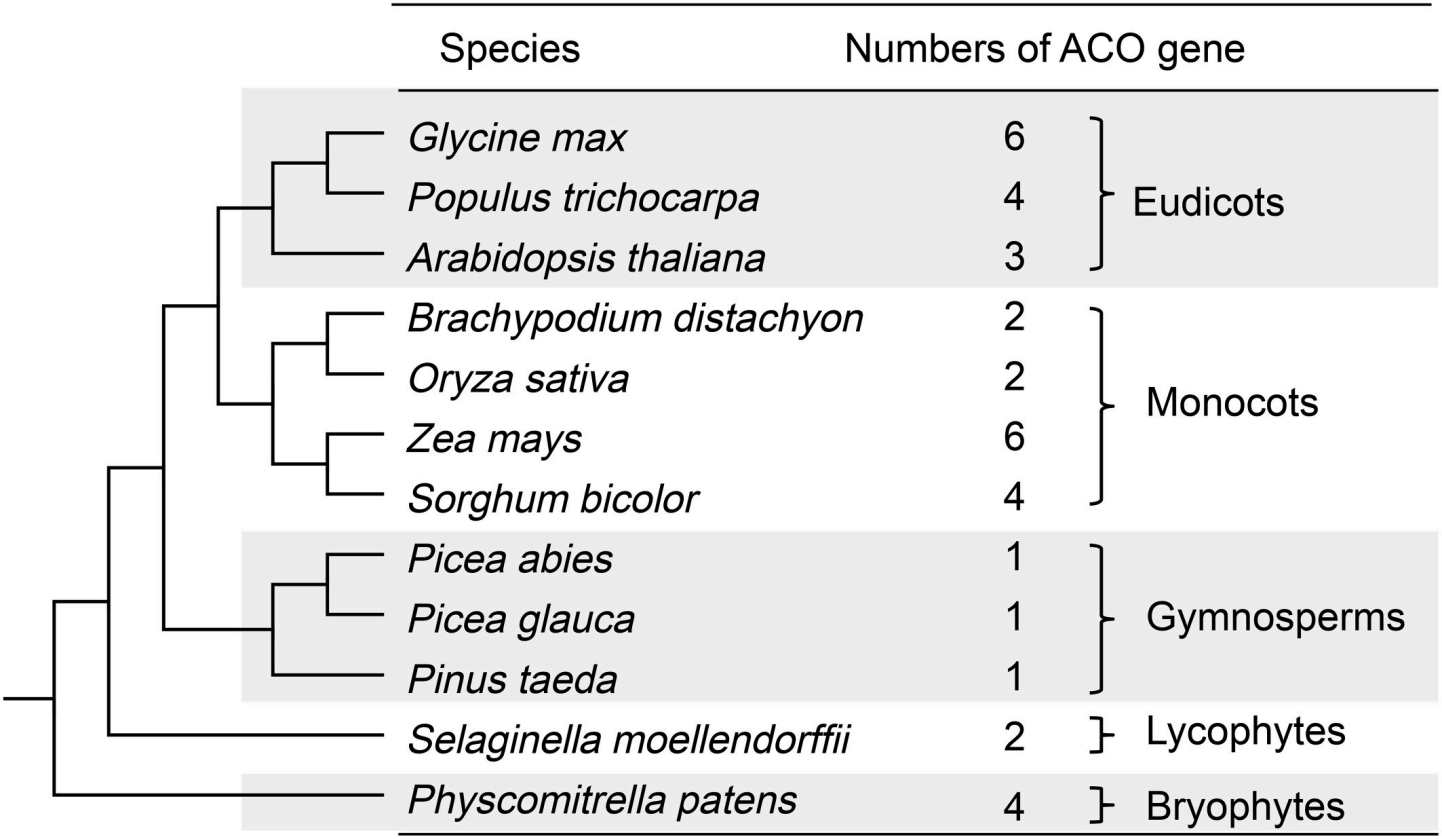
**Copy number variations of ACO genes in 12 land plant genomes**. The species relationships were redrawn according to Vanneste et al. (2014).

Copy number variations of the ACO genes in 12 land plant genomes were observed (Figure 1). The eudicots *G. max, P. trichocarpa*, and *A. thaliana* contained 6, 4, and 3 ACO genes, respectively. The monocots *B. distachyon, O. sativa, Z. mays*, and *S. bicolor* had 2, 2, 6, and 4 ACO genes, respectively. Each of three gymnosperms (*P. abies, P. glauca*, and *P. taeda*) contained only one ACO gene. *S. moellendorffii* and *P. patens* contained 2 and 4 ACO genes, respectively.

### Sequence features of ACO proteins

**Figure 2:**
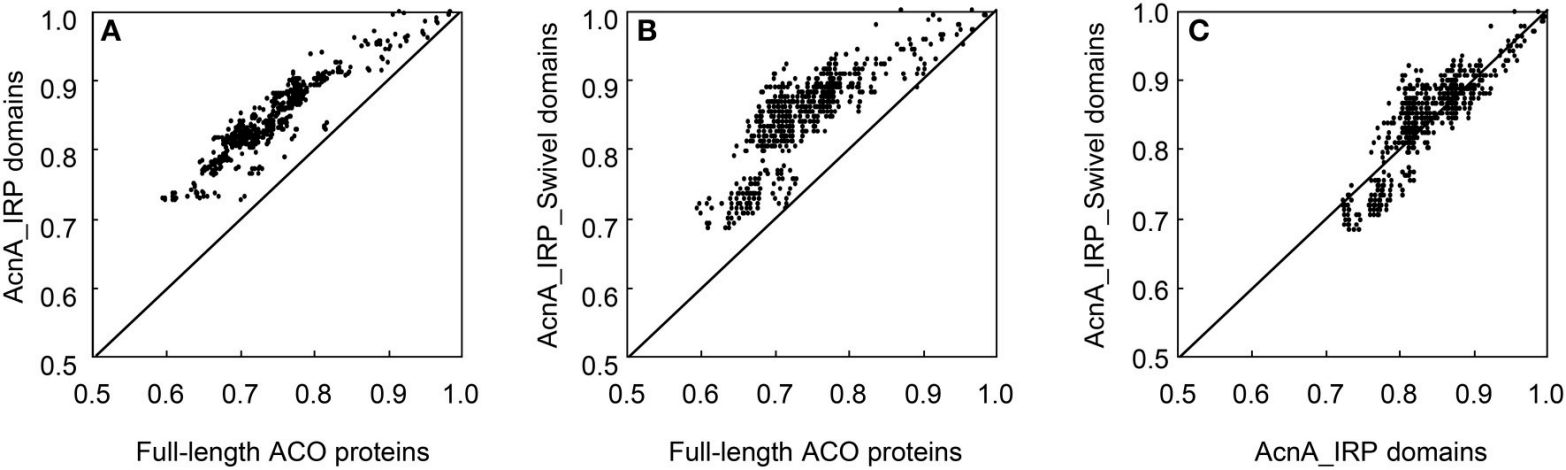
**Pairwise protein sequence identity plots for the full-length ACO proteins vs. AcnA_IRP domain (A)**, full-length ACO proteins vs. AcnA_IRP_Swivel domain **(B)**, and AcnA_IRP domain vs. AcnA_IRP_Swivel domain **(C)**.

The ACO proteins contained two distinct domains: AcnA_IRP domain, and AcnA_IRP_Swivel domain. Pairwise comparisons of the 36 ACO protein sequences revealed some notable features (Figure 2). The 36 full-length ACO proteins shared 59.6–98.5% pairwise sequence identity. The AcnA_IRP domains of 36 ACO proteins shared 72.5–99.7% pairwise protein sequence identity. The AcnA_IRP_Swivel domains of 36 ACO proteins shared 68.7–100.0% pairwise protein sequence identity. A pairwise comparison of protein sequences indicated a significant difference between the full-length protein and the AcnA_IRP domain protein sequences of 36 ACOs (paired sample *t*-test, *P* = 0.000). The AcnA_IRP domain protein sequences were much more conserved than full-length protein sequences (Figure 2A). We also observed that AcnA_IRP_Swivel domain protein sequences were more conserved than full-length protein sequences (paired sample *t*-test, *P* = 0.000, Figure 2B). Interestingly, our results showed a significant difference between the AcnA_IRP domain and the AcnA_IRP_Swivel domain protein sequence identities of 36 ACOs (paired sample *t*-test, *P* = 0.000). The AcnA_IRP_Swivel domain protein sequences were much more conserved than were the AcnA_IRP domain protein sequences (Figure 2C).

### Phylogenetic analyses and duplication history of the ACO gene family

**Figure 3:**
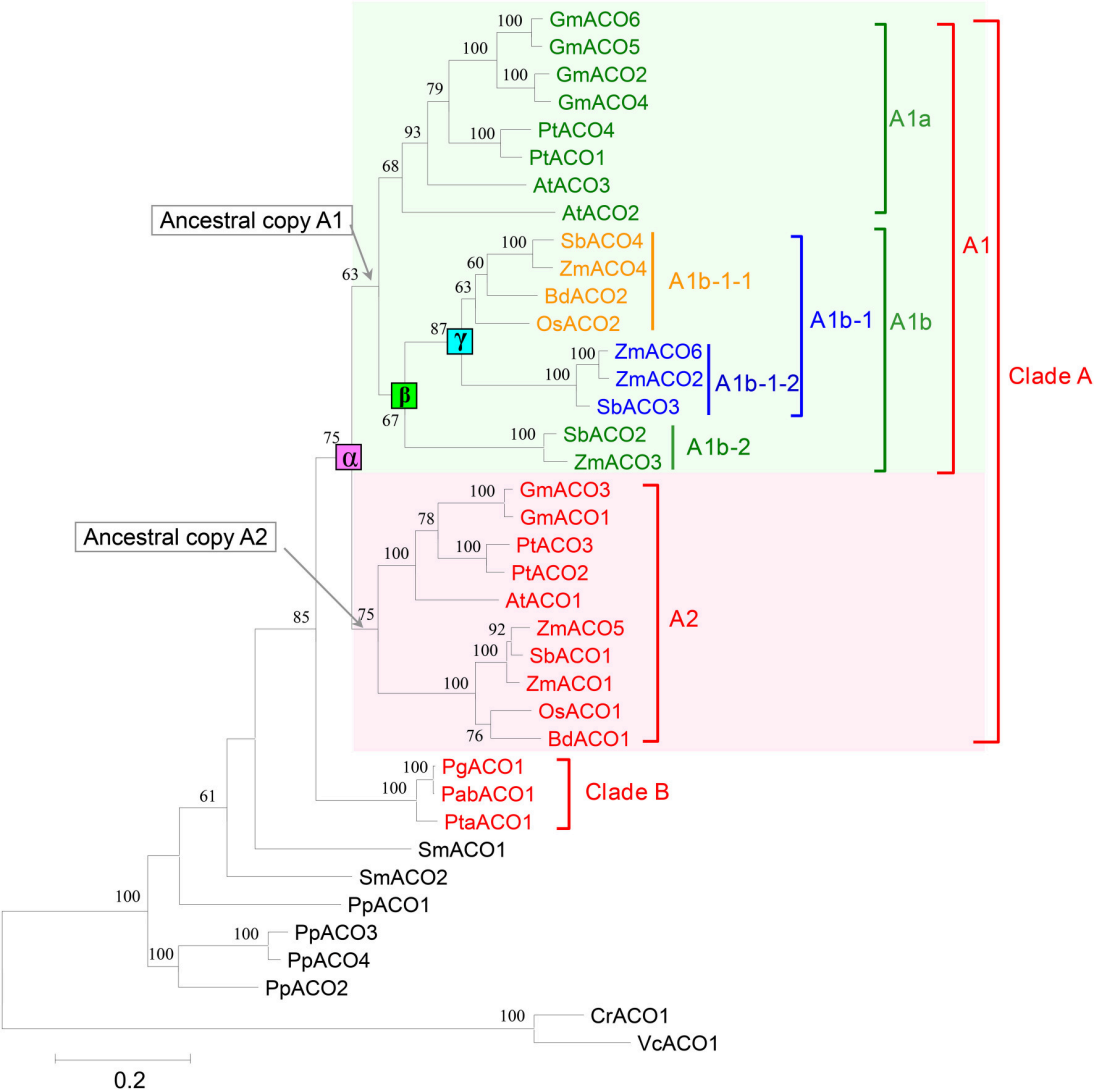
**Phylogenetic tree of 36 ACOs from 12 land plants**. Squares indicate three duplication events, annotated with α, β, and γ, respectively. Only bootstrap values >50% are shown.

In this study, we constructed a phylogenetic tree of the 36 ACO genes from 12 land plant species (Figure 3). The phylogenetic tree showed that all 27 ACO genes from the angiosperms were grouped into one clade (clade A in Figure 3), and three ACO genes from the gymnosperms were grouped into another clade (clade B in Figure 3). The 27 angiosperm ACO genes were divided into two subclades: A1 and A2. Each of clades A1 and A2 contained monocotyledon and dicotyledon ACO genes. We found that the monocotyledon clade A1b in clade A1 had complex phylogenetic relationships.

**Figure 4:**
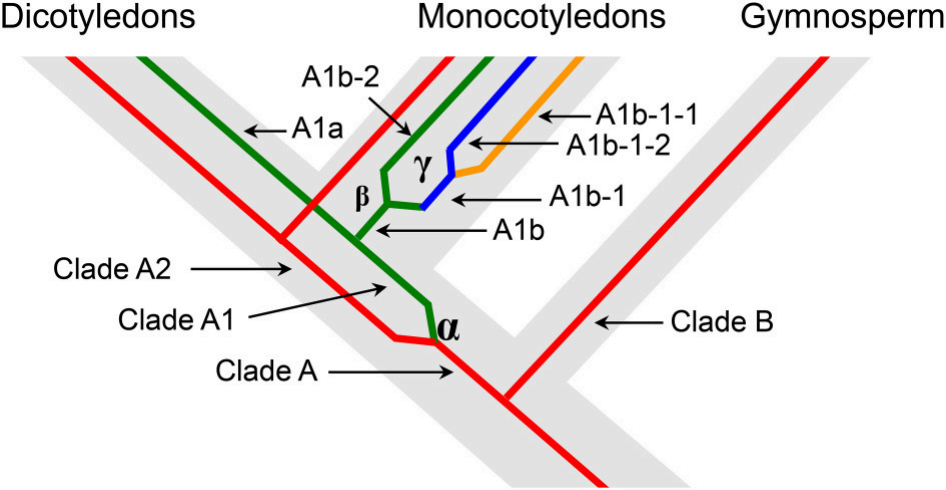
**Schematic representation of the duplication history of ACO genes in seed plants**. The designation of each clade corresponds to the Figure 3.

We predicted the duplication history of ACO genes in seed plants based on the phylogenetic tree of 36 ACO genes from 12 land plant species (Figure 4). The ancestor of seed plants may contain only one ACO gene because the ACOs from seed plants grouped into a single clade with high bootstrap support. This ancestral copy had been retained in gymnosperms, whereas a duplication event (α-duplication in Figures 3, 4) occurred on this ancestral copy in the ancestor of angiosperms. This duplication event resulted in two duplicate genes: Ancestral copies A1 and A2. These two duplicate genes were retained in monocotyledons and dicotyledons. We found that the ancestral copy A1 underwent two duplication events (β and γ-duplications in Figures 3, 4) in the monocotyledon lineage. The descendants originating from the β and γ-duplications were all retained in the linage of *Z. mays* and *S. bicolor*, whereas only one descendant was retained in the linage of *B. distachyon* and *O. sativa*. These three duplication events resulted in the expansion of ACO genes in angiosperms.

### Molecular evolution analyses

**Figure 5:**
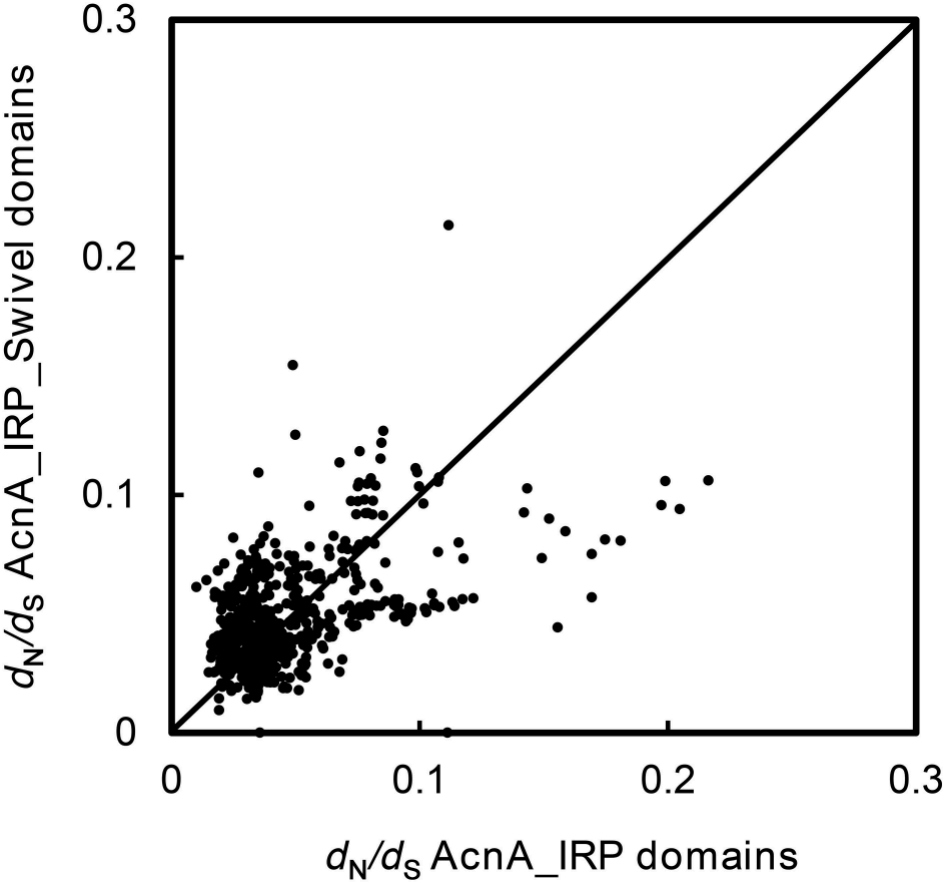
*****d***_**N**_/***d***_**S**_ plot for the AcnA_IRP domain vs. the AcnA_IRP_Swivel domain for each pair of 36 ACO genes**.

To test whether there were changes in selective pressure between the two distinct domains of ACO genes, we partitioned the 36 ACO sequences into AcnA_IRP domain (aliment position 220–711, Supplemental Figure S1) and AcnA_IRP_Swivel domain (aliment position 816–965, Supplemental Figure S1) regions. The ω values were calculated across all pairwise comparisons within each of the 36 ACO genes using the YN00 program in the PAML software package (Yang, 2007). The results suggested that no changes in selective pressure between the two domains were observed (Figure 5, paired sample *t*-test, *P* = 0.323).

Angiosperm ACO genes were divided into two clades: A1 and A2. To test whether there were changes in selective pressure between the two clades, we performed two branch-specific models using the PAML software package. The comparison of the one-ratio model with the two-ratio model using LRTs indicated that the two-ratio model was a significantly better fit than the one-ratio model (Table 1), suggesting significant differences in selective pressure between the two clades. Under the two-ratio model, the ω (= *d*_N_/*d*_S_) ratios for clades A1 and A2 were 0.13151 and 0.06925, respectively, indicating that the ACO genes in clade A1 were under more relaxed selection constraints.

**Table 1:** **Statistics for the detection of selection in angiosperm ACOs using branch specific models of PAML**.

**Model**	**Estimates of parameters**	**ln *L***	**2 *Δl***	***P***
One ratio	ω = 0.10704 for clade A	−33704.364917		
Two ratios	ω = 0.13151 for clade A1	−33651.801071	105.127692	<0.00001
	ω0 = 0.06925 for clade A2			

### Chromosomal distribution of the ACO gene in land plants

This study investigated the chromosomal locations of ACO gene in eight plant species; the exceptions were *P. abies, P. glauca, P. taeda*, and *S. moellendorffii*, whose genomes had not been assembled into chromosomes (Supplemental Figure S2). These 31 genes were dispersed in chromosomes, indicating that tandem duplication did not contribute to the expansion of plant ACO genes. We performed collinearity analysis of the chromosomal segments that harbored ACO genes to investigate whether segmental duplication contributed to the expansion of ACOs.

**Figure 6:**
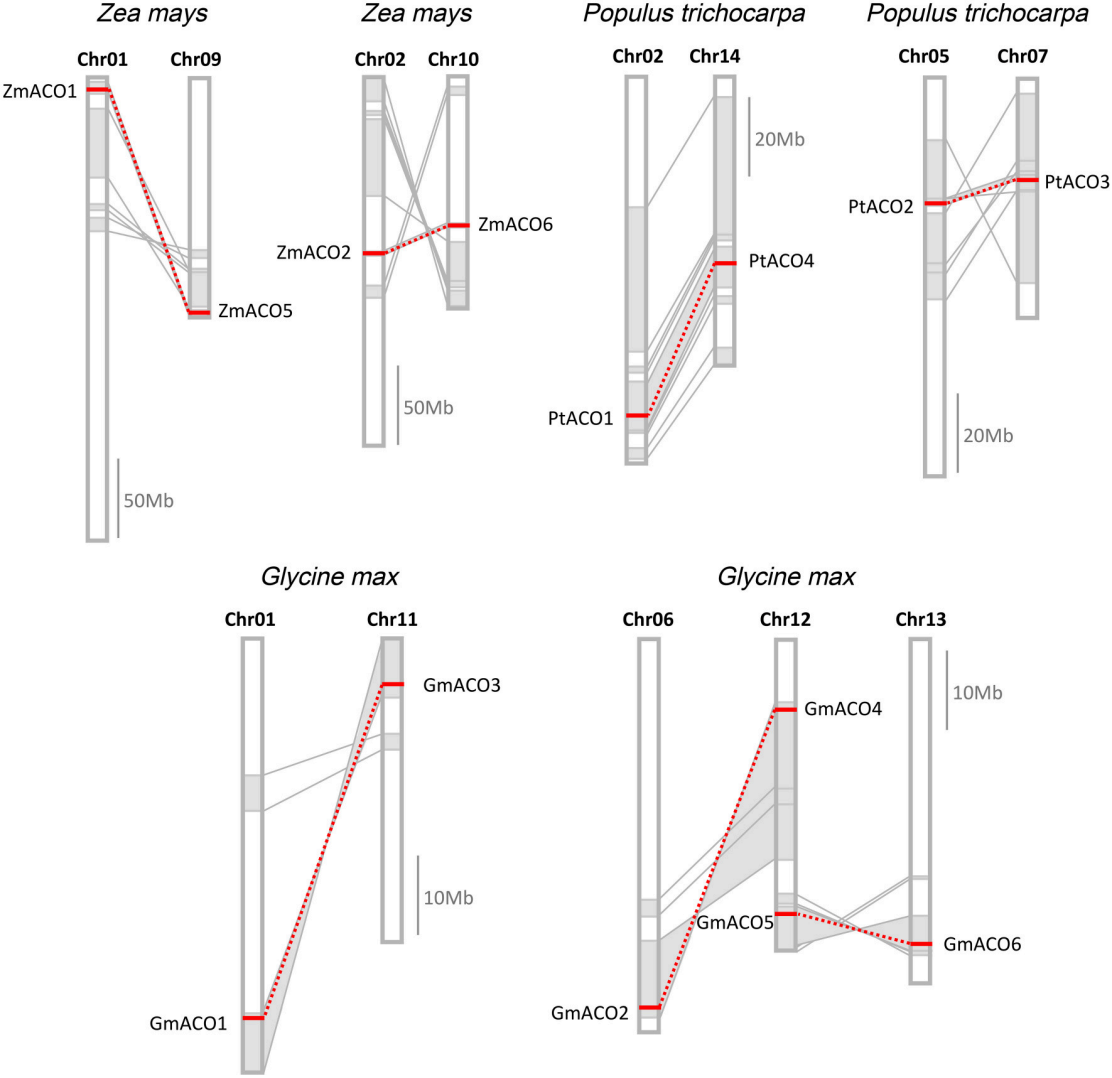
**Duplicate gene pairs created by segmental duplication in ***Z. mays, P. trichocarpa***, and ***G. max*****. Homologous genome blocks are shaded with gray and connected with lines. Duplicate gene pairs are connected with red dotted lines.

The result showed that, in *P. trichocarpa, G. max*, and *Z. mays*, seven duplicate pairs were located in paralogous blocks, indicating that they were created by segmental duplication (Figure 6). A whole-genome duplication (WGD) event in the Salicaceae (salicoid duplication) occurred about 60–65 million years ago (Tuskan et al., 2006). We found two duplicate pairs (*PtACO1/4* and *PtACO2/3*), each of which was located in a pair of paralogous blocks, indicating that the two duplicated gene pairs were created by a WGD event. The *G. max* genome has undergone two WGD events, occurring approximately 59 and 13 million years ago (Schlueter et al., 2004; Schmutz et al., 2010; Vanneste et al., 2014). We found three duplicate pairs in this study (*GmACO1/3, GmACO2/4*, and *GmACO5/6*); each duplicate pair was located in a pair of paralogous blocks created by the recent 13 Ma WGD event. This result may indicate that the three duplicate pairs in *G*. *max* were created by this most recent WGD event. We also found two duplicate pairs (*ZmACO1/5* and *ZmACO2/6)* in the *Z. mays* genome that were created by the WGD events. Three species (*P*. *trichocarpa, G*. *max*, and *Z*. *mays*) contained the most numerous ACO genes among the 12 land plants, and together they contained 16 ACO genes. Although 14 genes (seven duplicate pairs) out of 16 genes were created by segmental duplication in these species, no tandem duplication was found. This may indicate that segmental duplication was the preferred mechanism of the expansion of the ACO gene family.

### Gene structure of ACO genes in land plants

**Figure 7:**
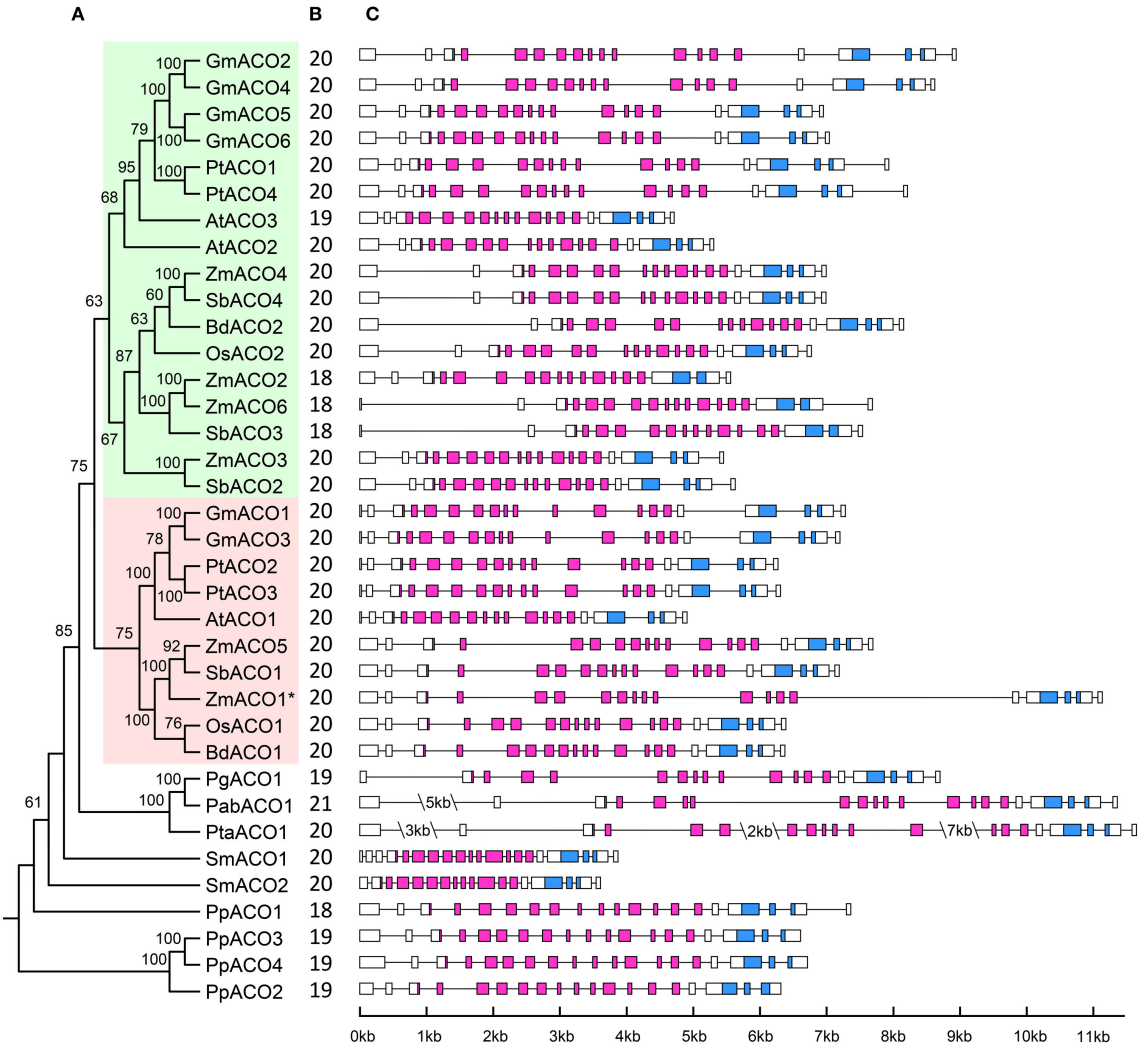
**Phylogenetic relationships and Gene structure of ACO gene family in land plants. (A)** The phylogenetic relationships of ACO genes, clade A1, and clade A2 are shaded in green and red, respectively. Putative pesudogene is marked with an asterisk. **(B)** Exon number of corresponding ACO genes. **(C)** Gene structure of ACO genes. AcnA_IRP domain and AcnA_IRP_Swivel domain are highlighted by the purple and blue boxes, respectively; introns are indicated as lines.

The exon-intron organization of the 36 ACO genes was examined to investigate the structural diversity of the ACO gene family in land plants (Figure 7). Although we did not find the start codon of *PgACO1* from the *P. glauca* genome, this gene contained full-length aconitase domain. Hence, *PgACO1* was included in the gene structure analysis as well. According to the results, plant ACOs contained 18–21 exons, and 72% of ACOs contained 20 exons. The exon number of genes in clade A2 was highly conserved; they all contained 20 exons. In contrast, the exon numbers of genes in clade A1 varied from 18 to 20. All ACO genes from eudicots in clade A1 contained 20 exons except for *AtACO3*, which contained only 19 exons. This is likely because the third intron of *AtACO3* was lost. Six of the nine ACO genes from monocots in clade A1 contained 20 exons, whereas the other three genes (*ZmACO2, ZmACO6*, and *SbACO3*) contained 18 exons. The ancestral gene of these three genes (*ZmACO2, ZmACO6, and SbACO3)* likely lost its 17th and 18th exons, which is in contrast to other ACOs from monocots in clade A1.

Intron length was variable within land plant ACOs. Some obvious intron length features were found in the ACOs from lycophytes (*S. moellendorffii*) and gymnosperms (*P. abies* and *P. taeda*). The whole intron lengths of the two ACO genes of *S. moellendorffii* were smaller than those of all other plant ACOs. In contrast, the intron lengths of ACO genes from two gymnosperm species (*P. abies* and *P. taeda*) were larger than those of all other plant ACOs. These intron lengths corresponded to the genome size of those species. The genome size of *S. moellendorffii* was approximately 212 mega base pairs, whereas the genome sizes of *P. abies* and *P. taeda* were both larger than 20 giga base pairs (Gonzalez-Martinez et al., 2007; Little et al., 2007; Nystedt et al., 2013). Additionally, *ZmACO1* and *ZmACO5* were a pair of segmental duplicated genes and should have had similar gene structures, but they did not; the 11th and 15th introns of *ZmACO1* were larger than those of *ZmACO5*. It probably because the *ZmACO1* was a pesudogene which under relaxed constrain.

### Expression divergence of ACO genes in land plants

**Figure 8:**
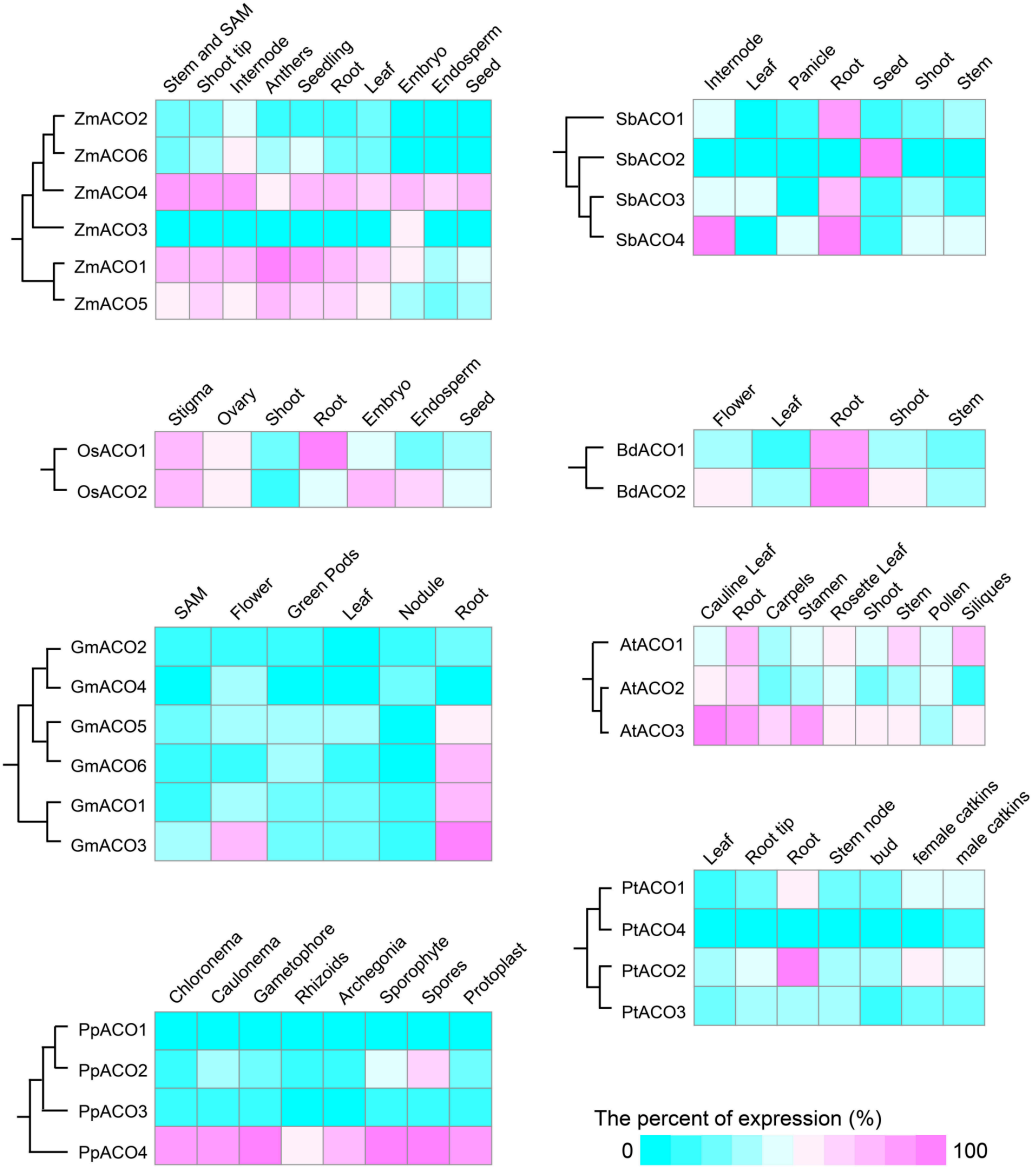
**The tissue-specific expression patterns of ACO genes**. Expression profiles for *Z. mays, S. bicolor, O. sativa, B. distachyon, G. max, A. thaliana, P. trichocarpa*, and *P. patens* are shown. Color indicates normalized expression level (the relative percent of the highest expression in each species). SAM is the abbreviation of shoot apical meristem.

To examine the functional diversification of ACO genes in land plants, we used RNA-seq data, microarray data, RT-PCR, and EST data to investigate the expression patterns. Each of three gymnosperms (*P. abies, P. glauca*, and *P. taeda*) contained only one ACO gene. All three gymnosperm ACO genes were expressed in the three tissues we examined (including stem, shoot, and root tissues). The RT-PCR results showed that the two ACO genes in *S. moellendorffii* were both expressed in stem, leaf, and root tissues (Supplemental Figure S3). The remainder of the 31 ACO genes in *P. patens* and angiosperm species showed expression divergence (Figure 8). *P. patens* contained four ACO genes. *PpACO4* showed a much higher expression level than did the other three *P. patens* ACO genes in all tissues examined. Similarly, *ZmACO4* displayed a higher expression level than the other 5 ACO genes of *Z. mays*.

Angiosperm ACO genes were divided into two clades: A1 and A2. Except for *Populus PtACO3*, all nine ACO genes in clade A2 showed high expression levels in the root tissues. However, eight of the 17 ACO genes in clade A1 did not show high expression levels in the root tissues. The pattern of high expression levels in the root tissues may be associated with the function of ACOs in nitrogen accumulation (Lancien et al., 1999; Carrari et al., 2003; Fernie et al., 2004). Additionally, tissue-specific expression patterns were detected in the ACOs. For example, some genes (e.g., *GmACO3, PtACO1*, and *PtACO3*) had relatively high expression levels in flower and root tissues, but low expression levels in other tissues. We observed that different expression patterns of the ACO gene family in land plants occurred not only among paralogs within one species but also among different tissues.

### *Cis*-acting element analysis of ACO gene family

**Figure 9:**
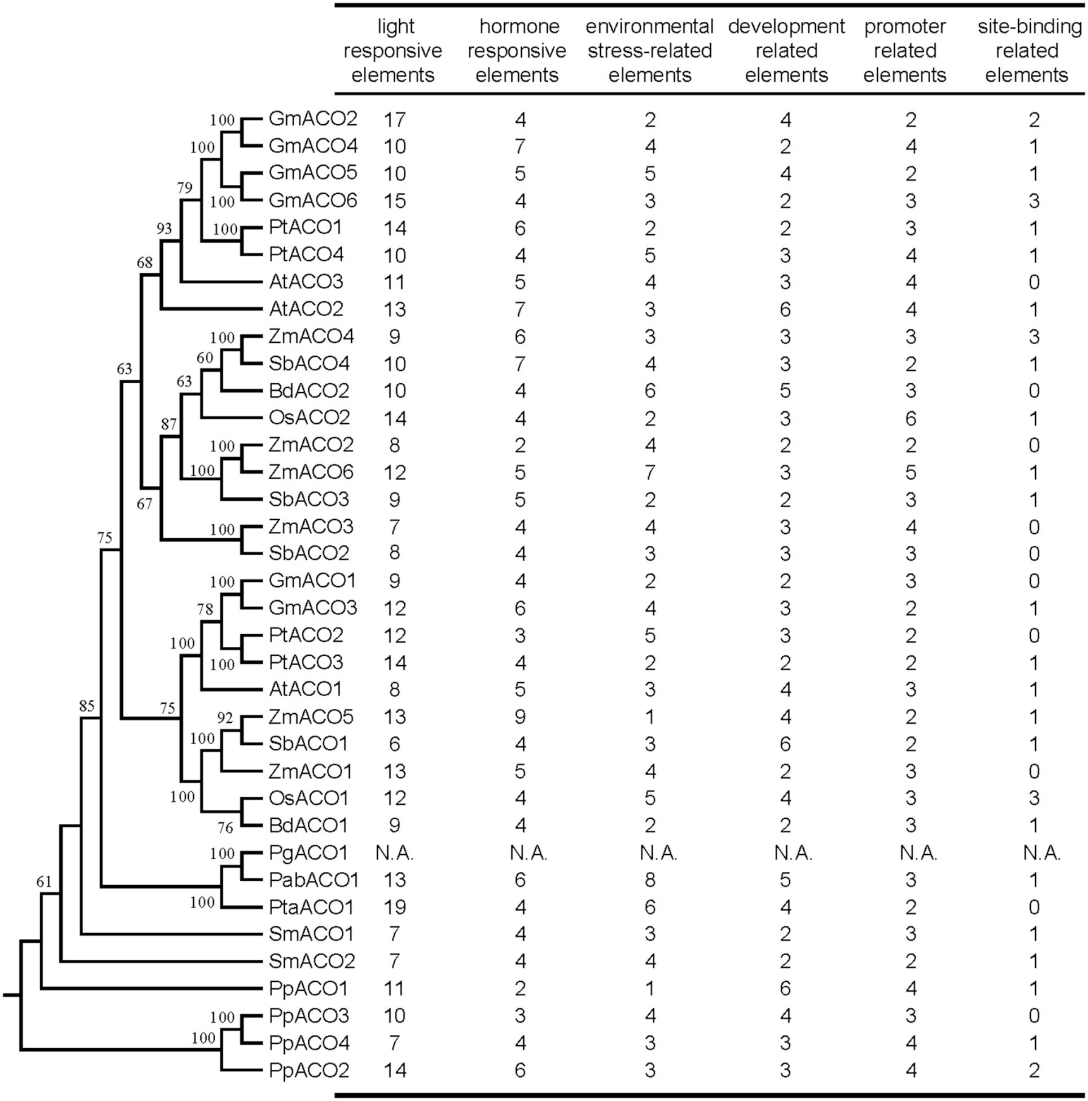
**Number of variations of ***cis***-acting elements in the promoter region of the ACO gene in land plants**. All *cis*-acting elements were divided into six categories according to their function. N.A. indicates that data are not available.

To understand the mechanism of transcriptional control, we identified the *cis*-acting elements in the promoter regions of the ACO genes using the PlantCARE program. We predicted the *cis*-acting elements of 1500 bp upstream promoter regions for each ACO gene (Figure 9); the exception was the *PgACO1* gene, for which we did not find the start codon from the *P. glauca* genome. Extensive divergence of *cis*-acting elements was displayed in the promoter regions of plant ACO genes (Figure 9 and Supplemental Table S3). For instance, the promoter region of *GmACO3* contained three *cis*-acting elements (CAT-box, CCGTCC-box, and ELI-box3), which were absent in the other 5 ACOs of *G. max*. Five *cis*-acting elements (including circadian, EIRE, ABRE, 3-AF1 binding site, and chs-Unit 1 m1) were detected in *PpACO4* but were not present in the other three *P. patens* ACO genes. This divergence of *cis*-acting elements may be responsible for the expression divergence of the plant ACO gene family.

The *cis*-acting elements (e.g., G-box, G-Box, Sp1, Box4, BoxI, GAG-motif, etc.) that function in light responsiveness (Supplemental Table S3) were most abundant in the promoter regions of ACO genes, indicating that plant ACO genes might participate in light regulatory pathways. Additionally, promoters of ACO genes that also harbored *cis*-acting elements responded to phytohormones such as methyl jasmonate (MeJA), abscisic acid (ABA), auxin, ethylene, salicylic acid (SA), and gibberellin. We found that each promoter region of ACO genes in the angiosperm A2 clade (Figure 3) contained two *cis*-acting elements responding to MeJA, whereas the promoter regions of three ACO genes (*OsACO2, SbACO2*, and *ZmACO2*) in angiosperm clade A1 did not (Figure 3). These results indicate that plant ACOs may play a role in phytohormone responsiveness.

## Discussion

Aconitases in plants play essential roles in the TCA and glyoxylate cycles. We performed a comprehensive analysis of the ACO family of land plants. The duplication history analysis showed that three duplication events had occurred in the ACO family of angiosperms: One in the angiosperm ancestral species, and the other two were monocot-specific. The results of this study showed that duplication contributed to the expansion of the ACO gene family in angiosperms. No tandem duplication was observed in the ACO gene family, whereas segmental duplications were detected in *P. trichocarpa, G. max*, and *Z. mays*. Segmental duplication seems to be the primary gene expansion mechanism of the ACO gene family in these three species. Additionally, angiosperm clades A1 and A2 resulted from α-duplication (Figure 3), and different selective pressures were detected on those two clades. The molecular evolution analyses showed that the genes in clade A1 were under more relaxed selection constraints, whereas genes in clade A2 were under stronger purifying selection. We inferred that the ACO genes in clade A2 are more conserved than genes in clade A1. Gene structure analysis showed that the genes in clade A2 had more conserved exon/intron organization than did genes in clade A1. The expression profiles were also in accord with this conjecture. For example, all nine ACO genes in clade A2, except for *PtACO3*, were highly expressed in the root tissues, whereas the expression of genes in clade A1 diverged in root tissues.

Based on the phylogenetic tree and chromosomal distribution analysis, we identified seven recent duplicate gene pairs (including *PtACO1/4, PtACO2/3, GmACO1/3, GmACO2/4, GmACO5/6, ZmACO1/5*, and *ZmACO2/6*) from three species. Except for one gene pair (*ZmACO1/5*), each duplicate gene pair was grouped together. This is likely because *ZmACO1* was a pseudogene with relaxed constraints and accumulated more substitutions than *ZmACO5*. We then analyzed the expression profiles of those duplicate gene pairs and found some intriguing features. Both of the duplicated genes in the duplicate gene pairs *GmACO2/4* and *GmACO5/6* showed similar expression levels in the same tissues. However, for the remaining five duplicate gene pairs, two duplicated genes displayed different expression patterns. For example, for the duplicate gene pair *GmACO1/3, GmACO3* was highly expressed in flower tissue, whereas *GmACO1* was not. The *cis*-acting element analysis showed that *GmACO3* had three distinctive *cis*-acting elements (including CCGTCC-box) that did not exist in *GmACO1*. The *cis*-acting element CCGTCC-box is related to meristem-specific activation, which may be responsible for the expression divergence of the duplicate gene pair *GmACO1/3*.

Light is a regulator of the TCA cycle which plays a central role in the respiratory chain (Igamberdiev and Gardestrom, 2003; Fernie et al., 2004; Nunes-Nesi et al., 2007). Interestingly, we found there were many light responsiveness *cis*-acting elements in the promoter regions of the ACO genes. For example, there are 13 and 11 light-responsiveness *cis*-acting elements in the promoters of *AtACO2* and *AtACO3*, respectively (Figure 9). *AtACO2* and *AtACO3* showed relatively high expression levels in the cauline leaf tissues (Figure 8). We inferred that, as an essential enzyme of the TCA cycle, the aconitases are regulated by light which is also the case for other enzymes in the Krebs cycle are (Fernie et al., 2004). Although no direct evidence of light regulating the aconitases function has yet been reported, a previous study showed that the ACO mutant of the wild tomato species had decreased aconitase activity but an elevated photosynthesis rate (Carrari et al., 2003). Considerable evidence supports the involvement of plant ACOs in the oxidative stress response (Navarre et al., 2000; Arnaud et al., 2007; Moeder et al., 2007). And MeJA and ABA are important hormones for oxidative homoeostasis. The results of the *cis*-acting element analyses in this study showed that all ACO genes from land plants contained *cis*-acting elements responding to MeJA and/or ABA. Additionally, the expression profiles showed that many ACOs had a high expression levels in root tissues (e.g., *OsACO1, GmACO3, BdACO2*, etc.) These results indicate that plant ACOs may be involved in oxidative responsiveness by responding to hormones.

Our study showed that all ACOs of land plants were cytosolic aconitases and that no mitochondrial isoform was detected in those genomes. However, the ACO genes from two green algae species both belonged to the mitochondrial isoform. We performed a BLAST search in the NCBI nucleotide database (data not shown) to investigate whether algae contained cytosolic ACOs. The results showed that there was no cytosolic aconitase in charales, red algae, or glaucophytes. Although it was widely assumed that land plants originated from streptophyte green algae (Lewis and McCourt, 2004; Becker and Marin, 2009), the phylogenetic analysis showed that the ACOs in land plants and green algae did not form a monophyletic group. And previous study inferred the cytosolic aconitases of land plants may have originated from eubacteria (Schnarrenberger and Martin, 2002). Taken together, these results indicate that the ancestral cytosolic ACO of land plants may have been independently acquired rather than inherited from a green algae ancestor. Further investigation is required to elucidate the origin of land plant ACO genes.

## Author contributions

HY conceived the project. YW performed the experiments. QY and YL analyzed the data. QY and HY wrote prepared the manuscript.

## Funding

This study was supported by grant from the National Natural Science Foundation of China (NSFC 31270641).

### Conflict of interest statement

The authors declare that the research was conducted in the absence of any commercial or financial relationships that could be construed as a potential conflict of interest.
