# Supplementary material for: Molecular Evolution and Expression Divergence of the Aconitase (ACO) Gene Family in Land Plants
Source: Front Plant Sci. 2016 Dec 12;7:1879. doi: 10.3389/fpls.2016.01879 (PMC5149538; doi:10.3389/fpls.2016.01879)

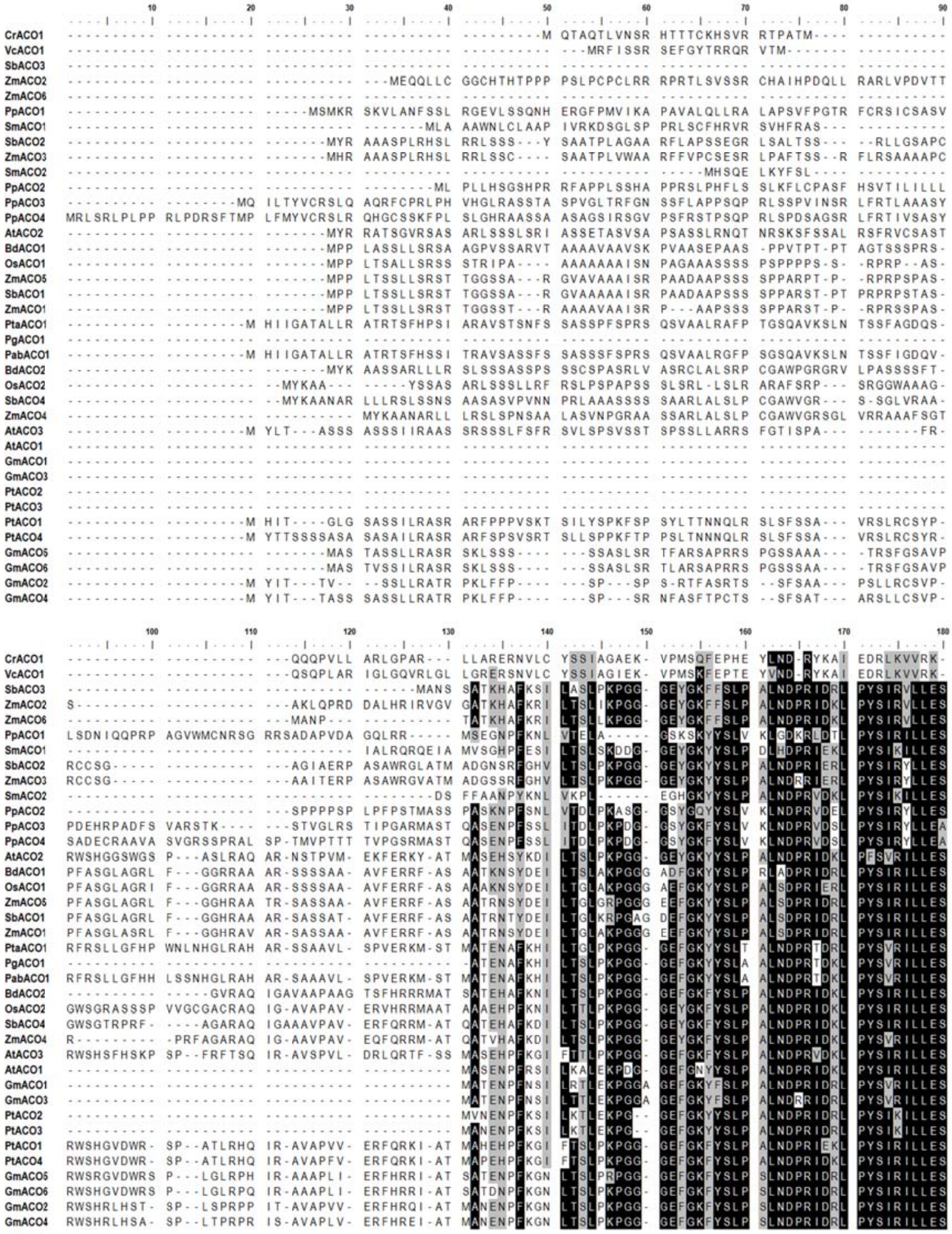

Supplemental Figure S1. **Sequence alignment of ACOs in land plants.** Residues conserved in all ACO proteins within a 70% threshold are shaded.

Supplemental Figure S1. (continued).

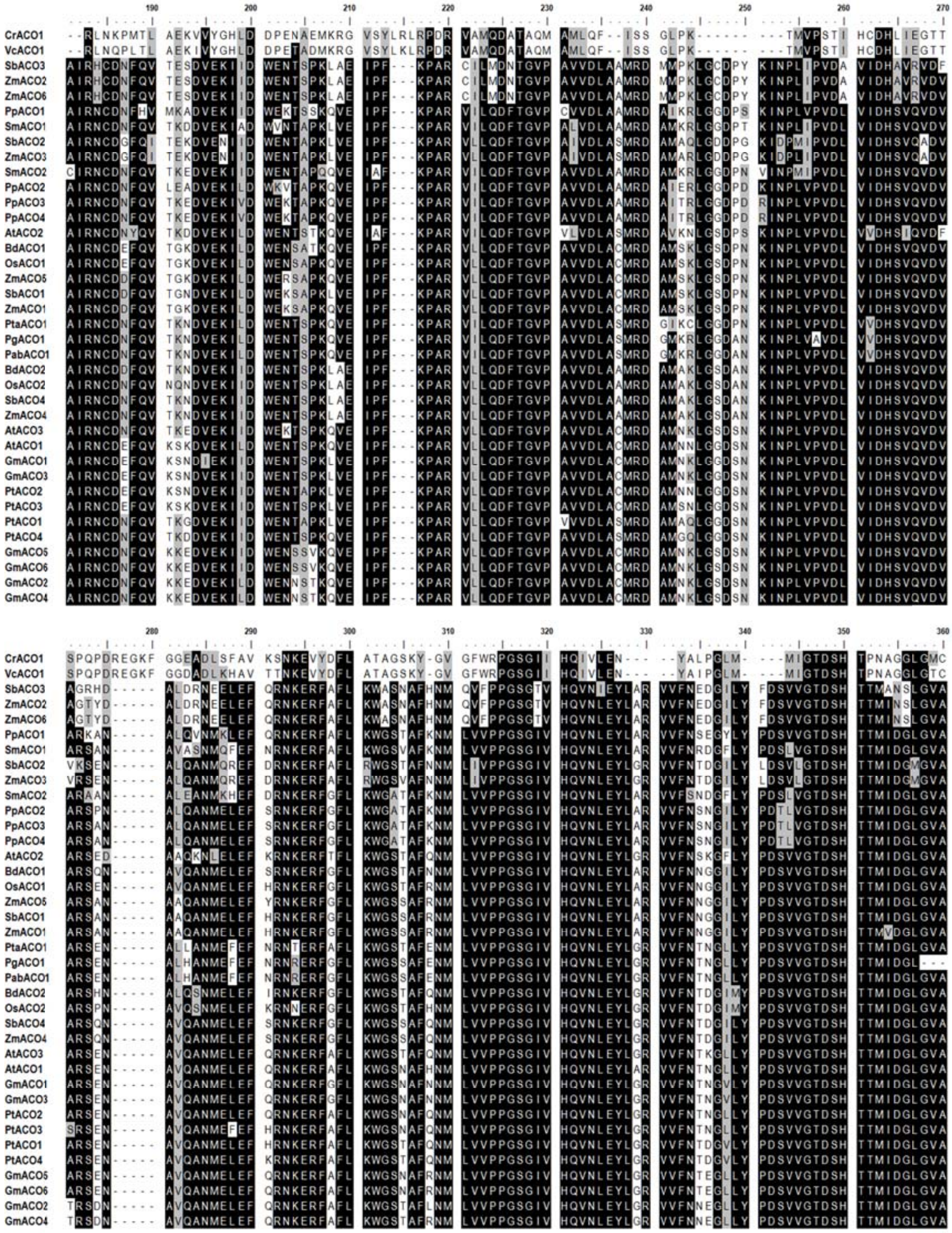

Supplemental Figure S1. (continued).

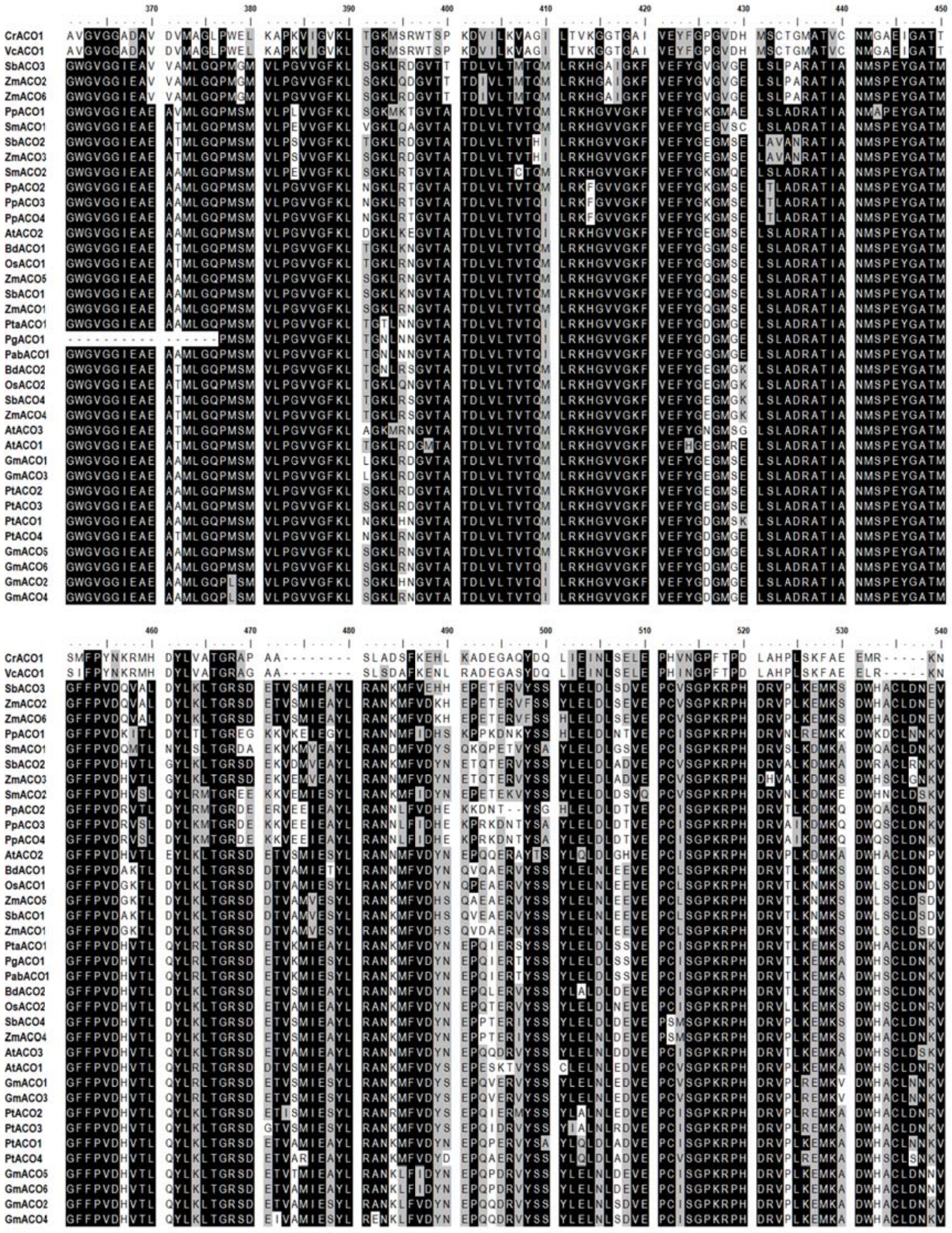

Supplemental Figure S1. (continued).

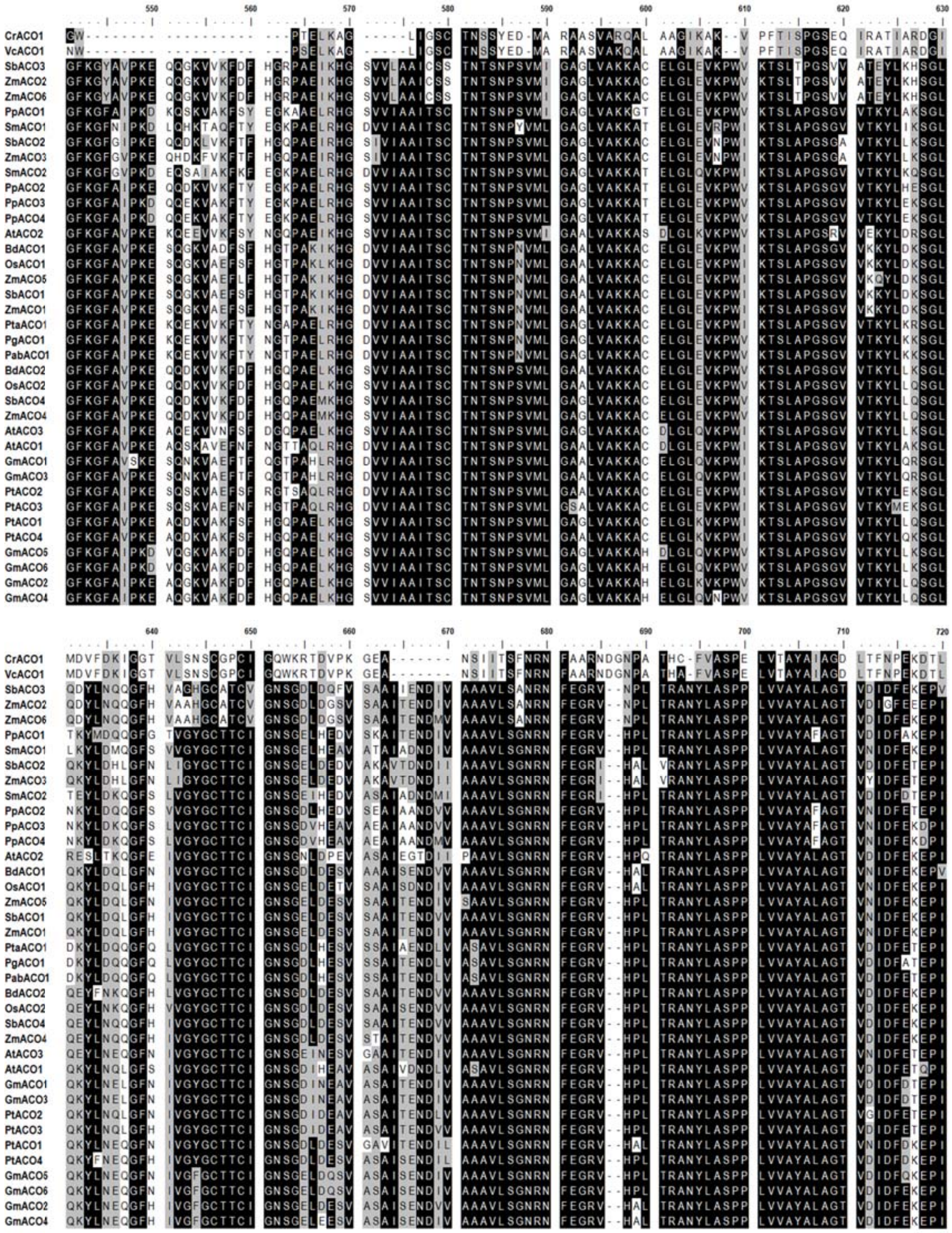

Supplemental Figure S1. (continued).

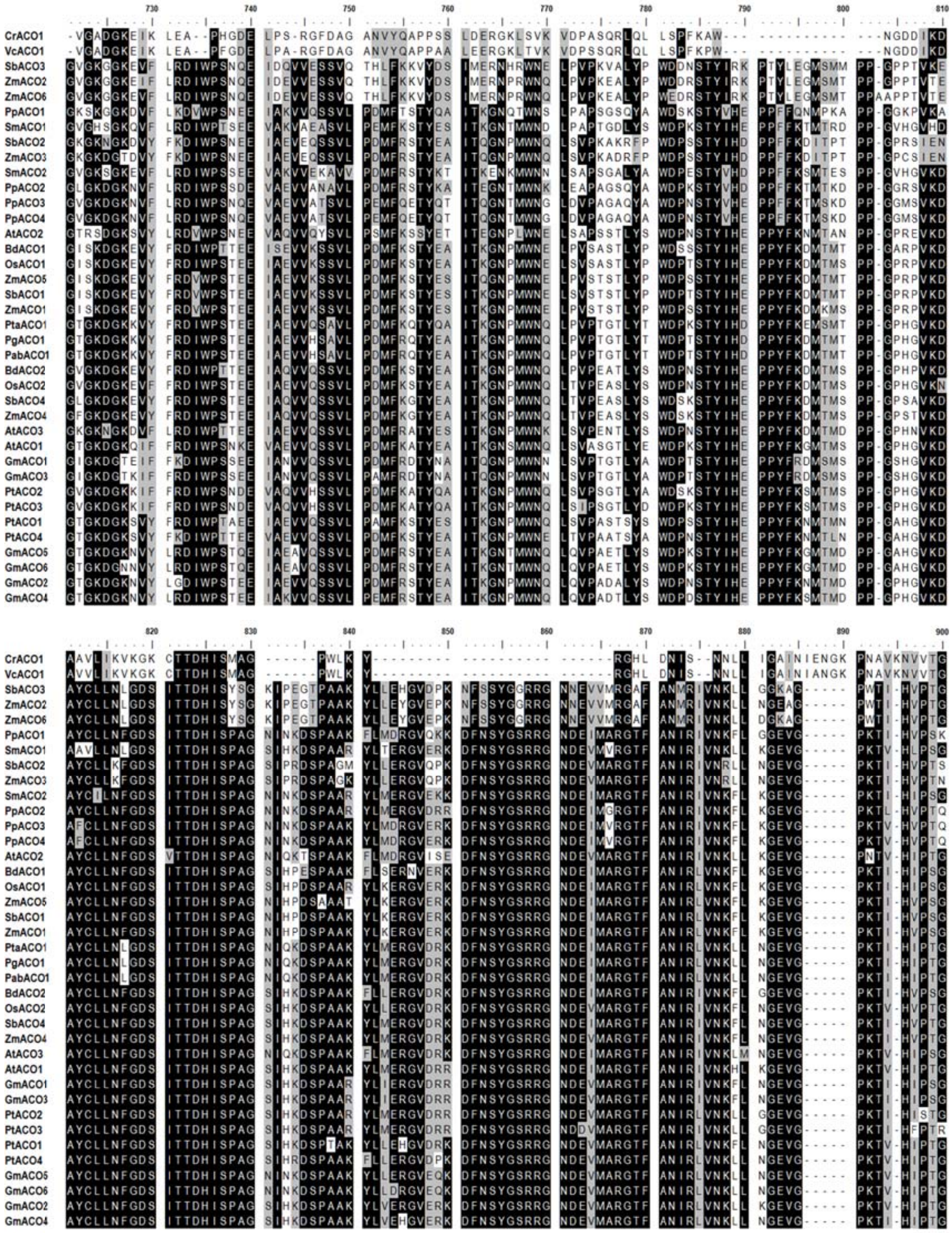

Supplemental Figure S1. (continued).

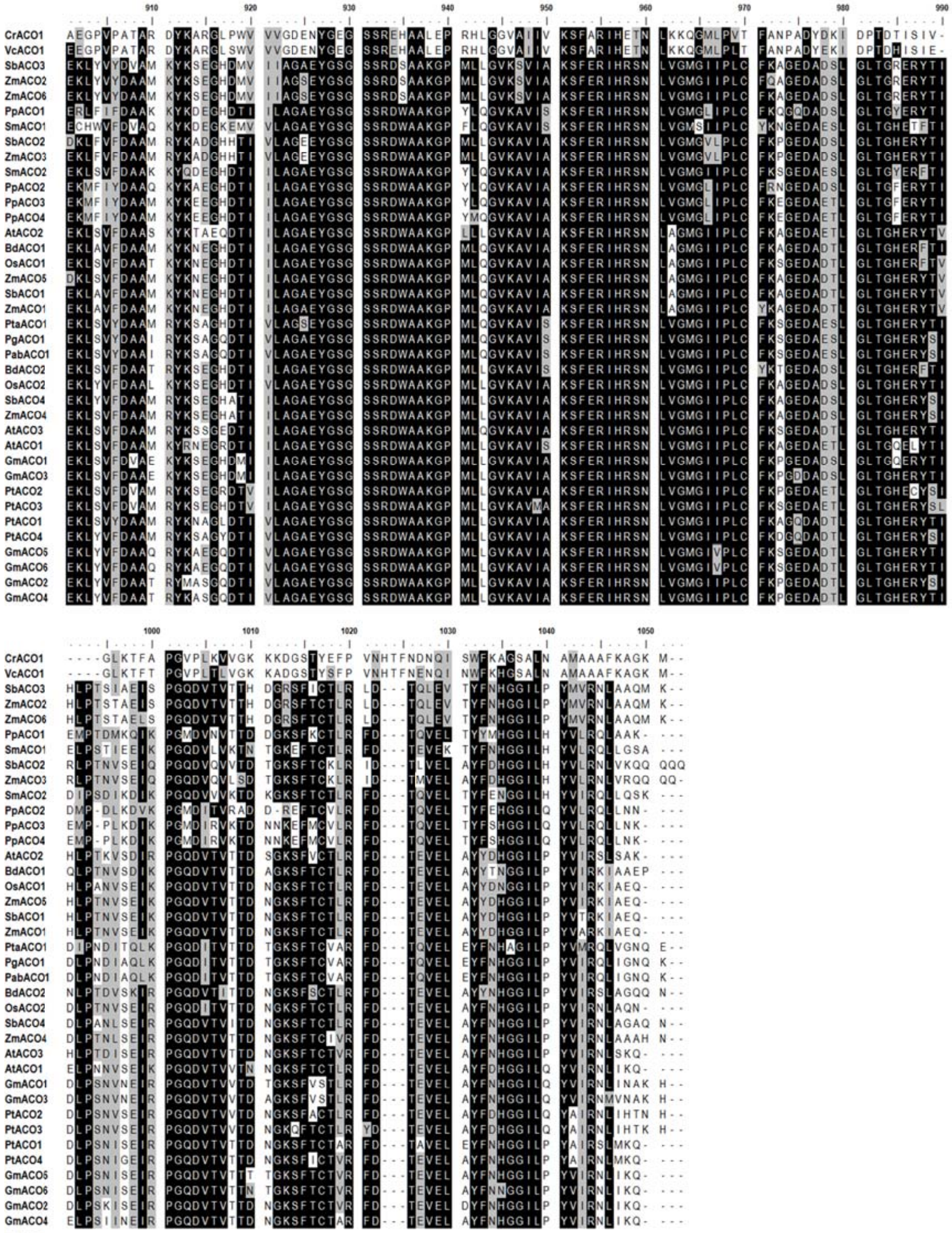

Supplement: Supplementary file 1 [file Image1.PDF]
