# Supplementary material for: Molecular Evolution and Expression Divergence of the Aconitase (ACO) Gene Family in Land Plants
Source: Front Plant Sci. 2016 Dec 12;7:1879. doi: 10.3389/fpls.2016.01879 (PMC5149538; doi:10.3389/fpls.2016.01879)

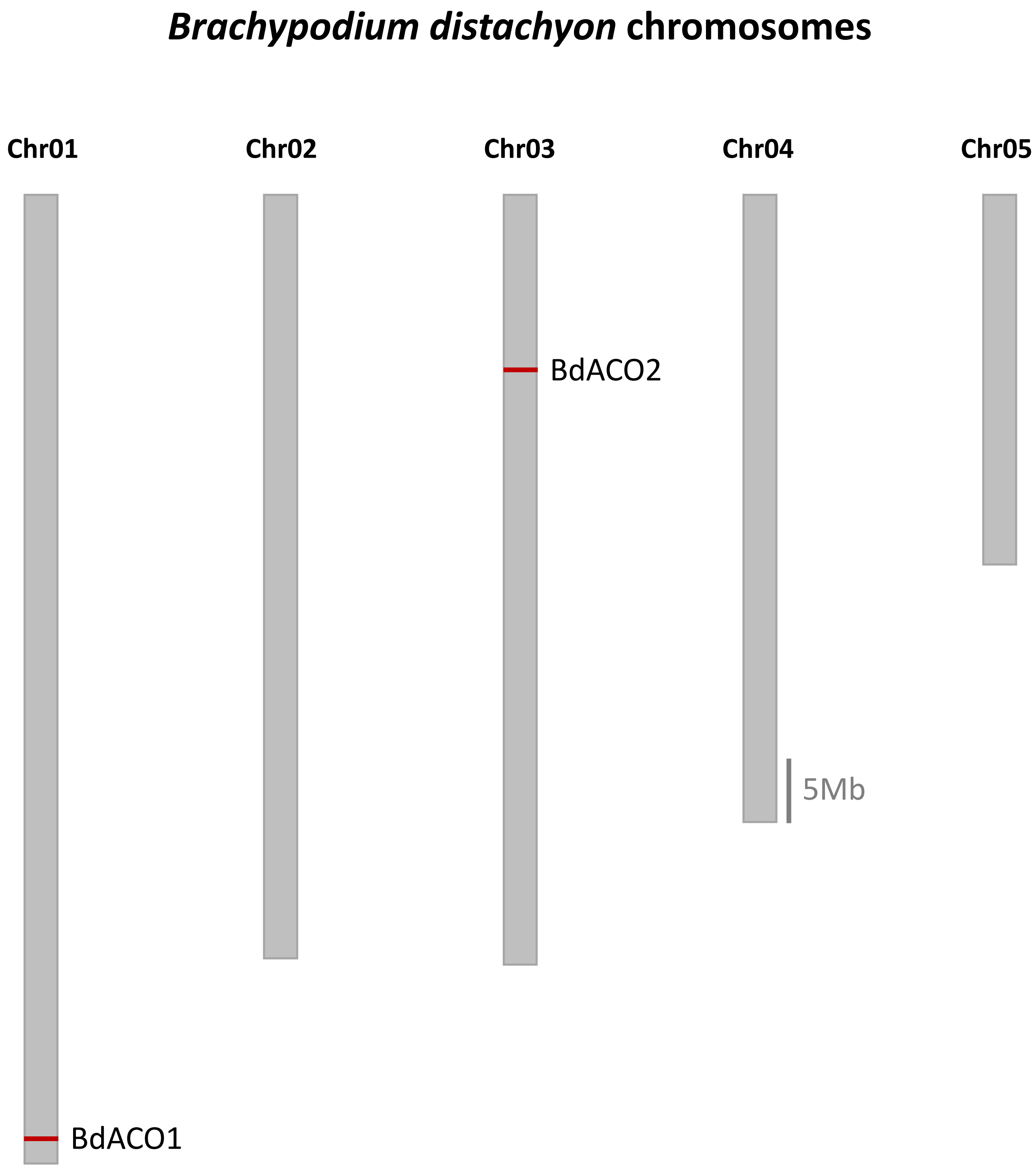

Supplemental Figure S2. Genomic localization of ACO genes. Schematic diagrams are only drawn in the chromosome-assembled plant genomes. ACO genes were indicated by red lines.

Supplemental Figure S2. (continued).

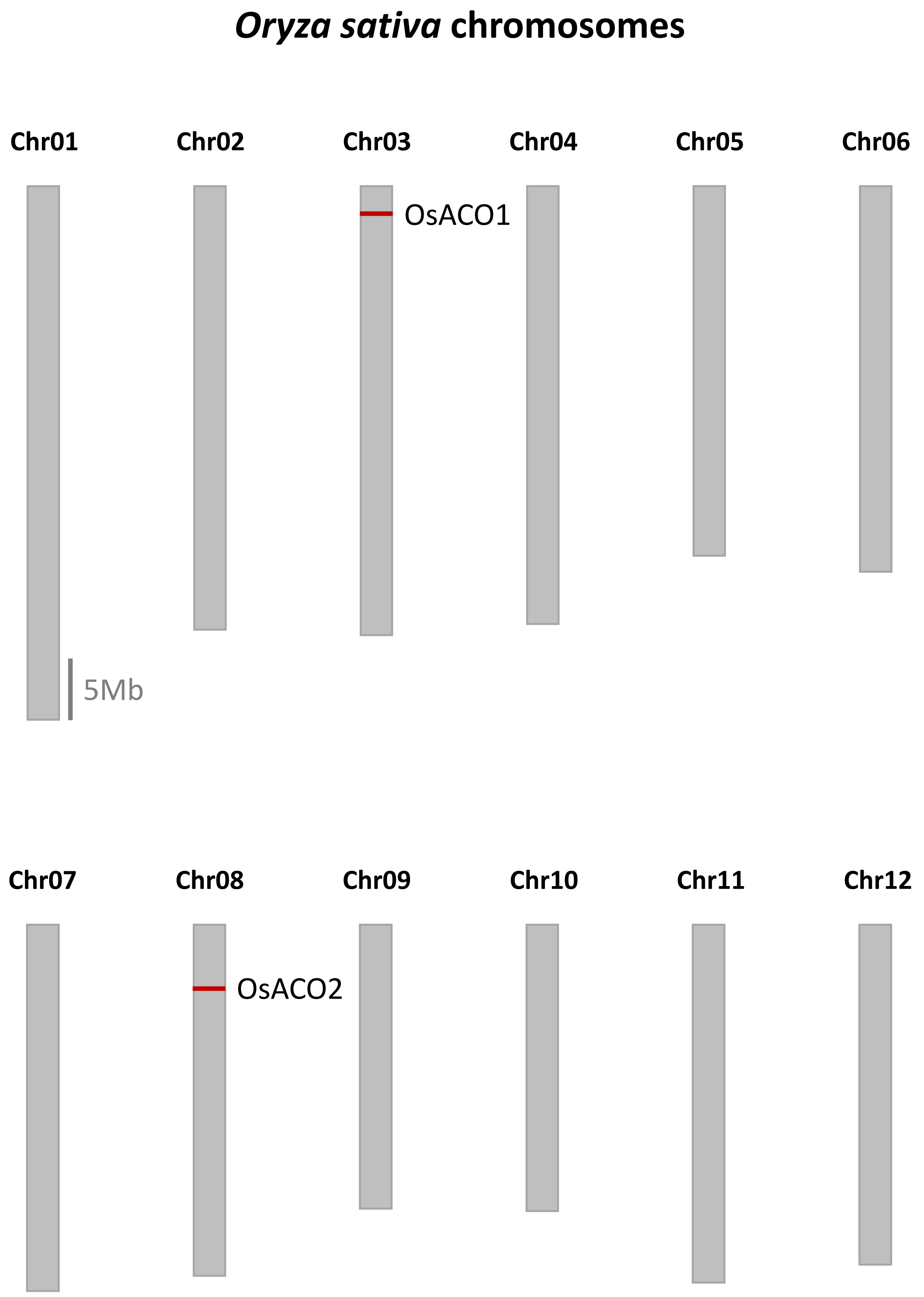

Supplemental Figure S2. (continued).

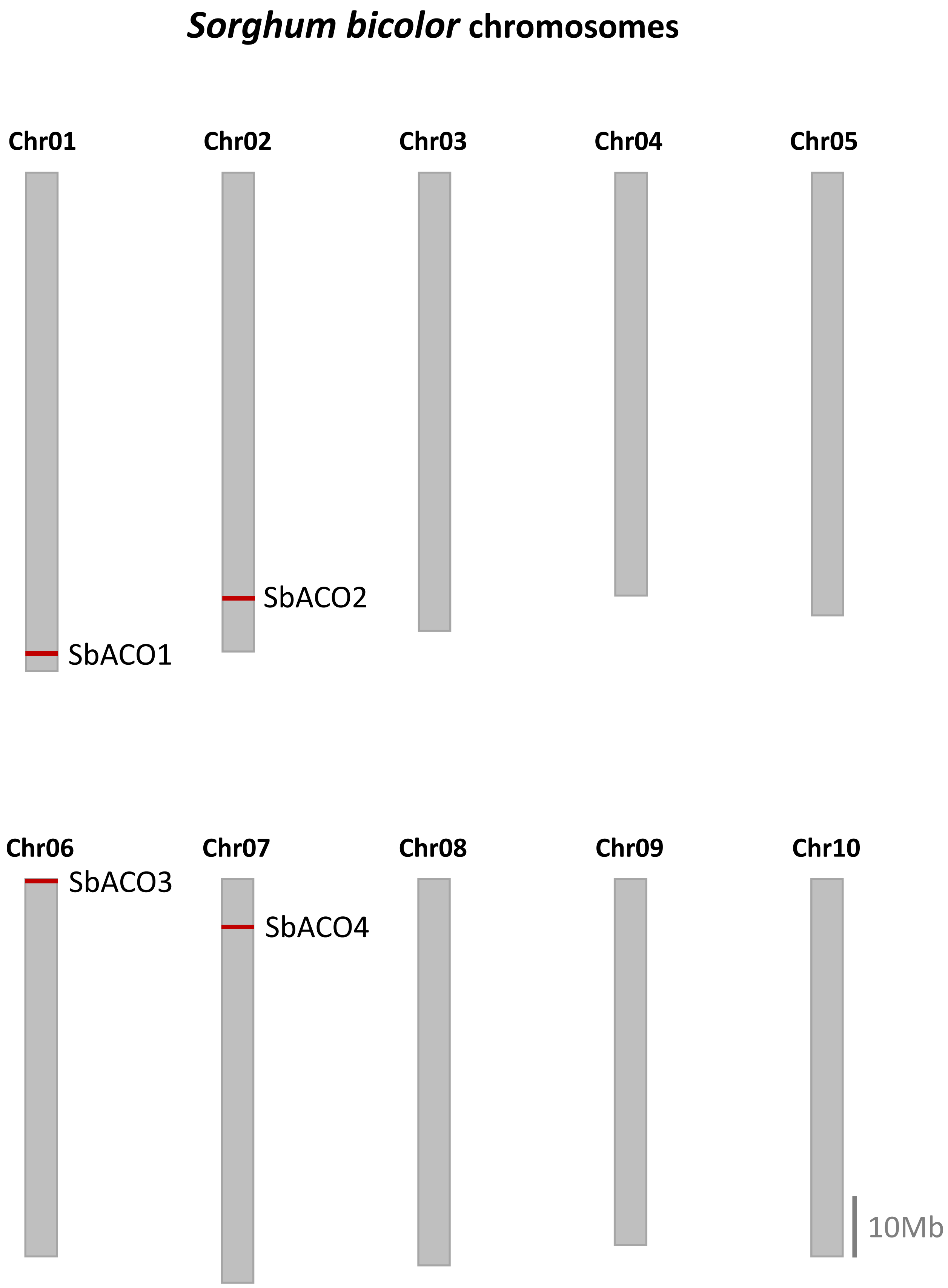

Supplemental Figure S2. (continued).

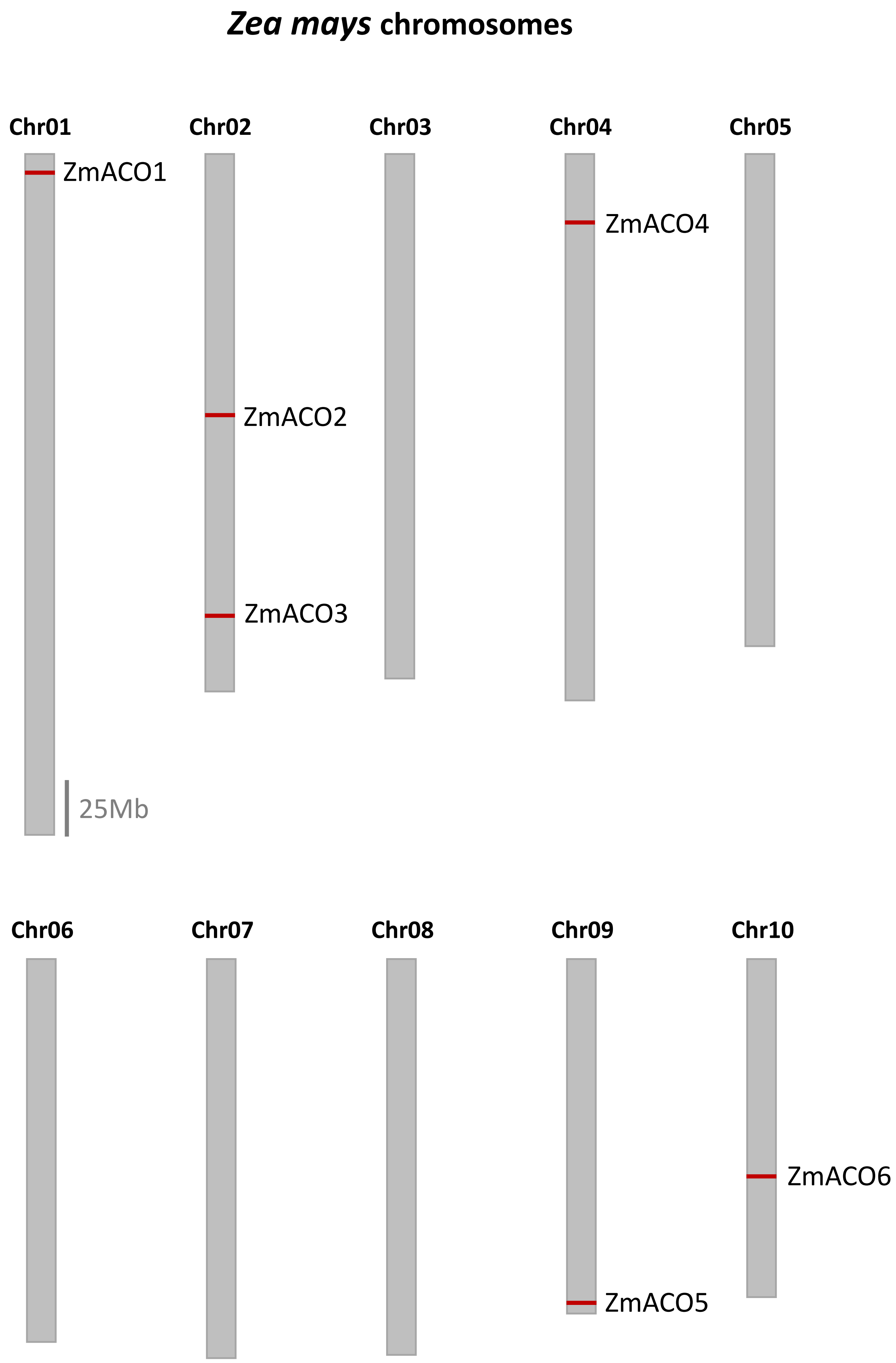

Supplemental Figure S2. (continued).

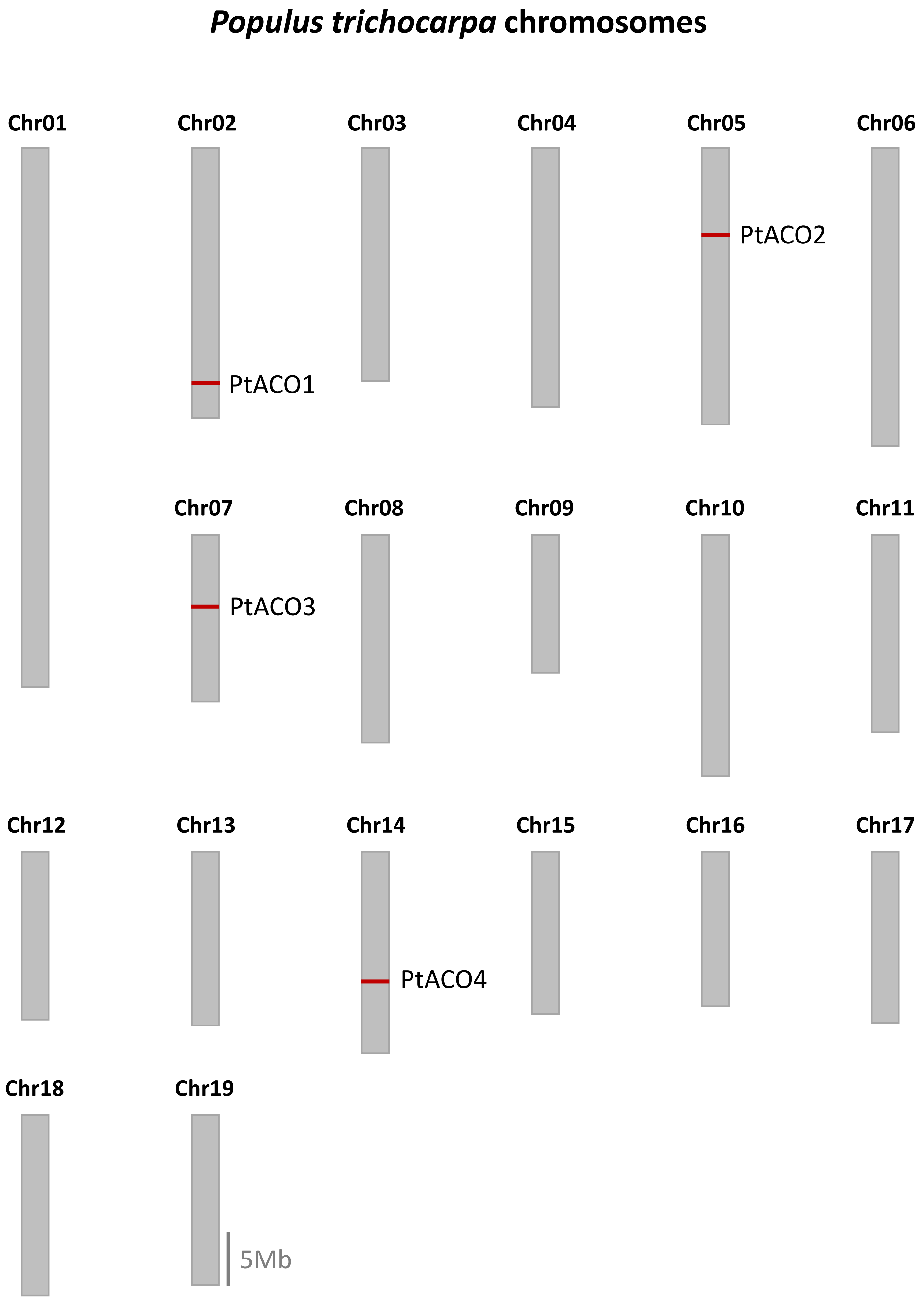

Supplemental Figure S2. (continued).

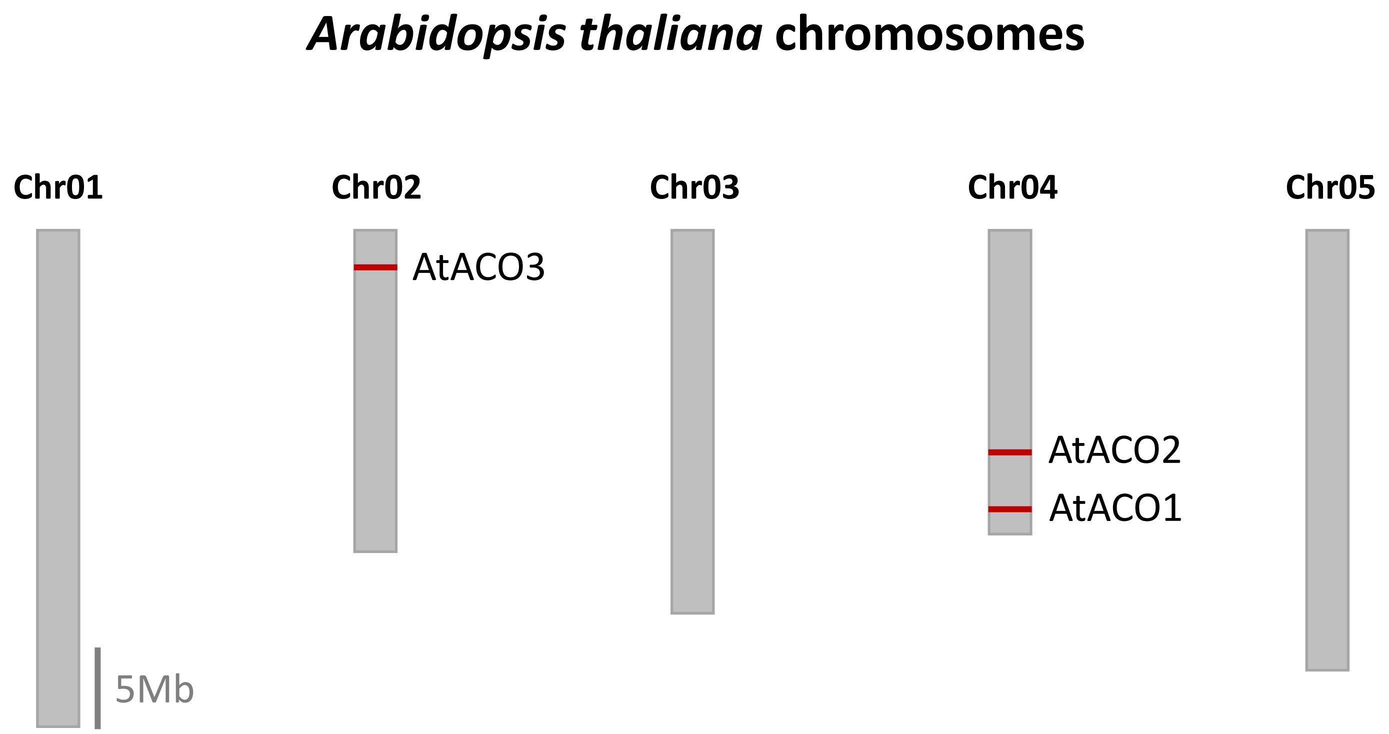

Supplemental Figure S2. (continued).

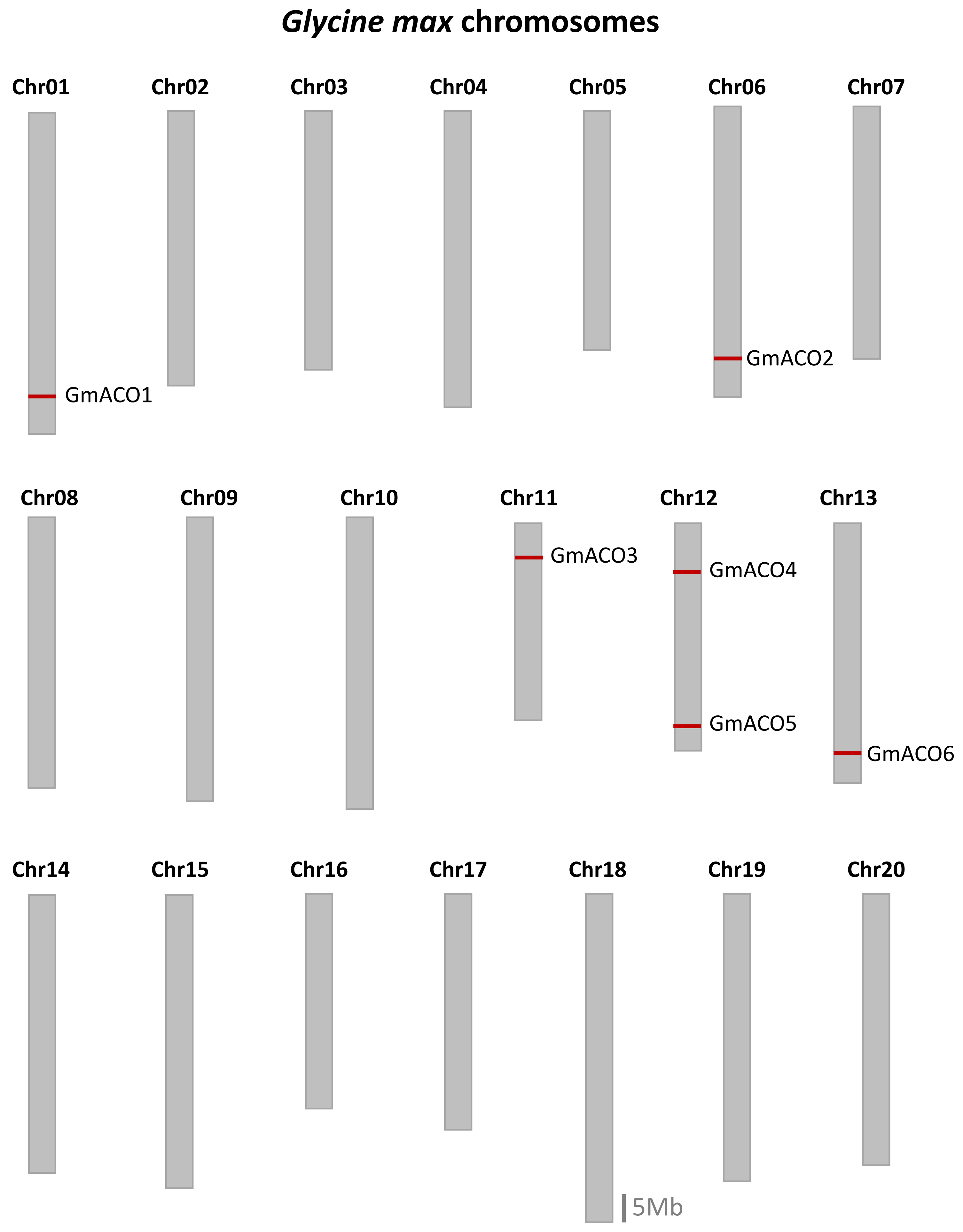

Supplemental Figure S2. (continued).

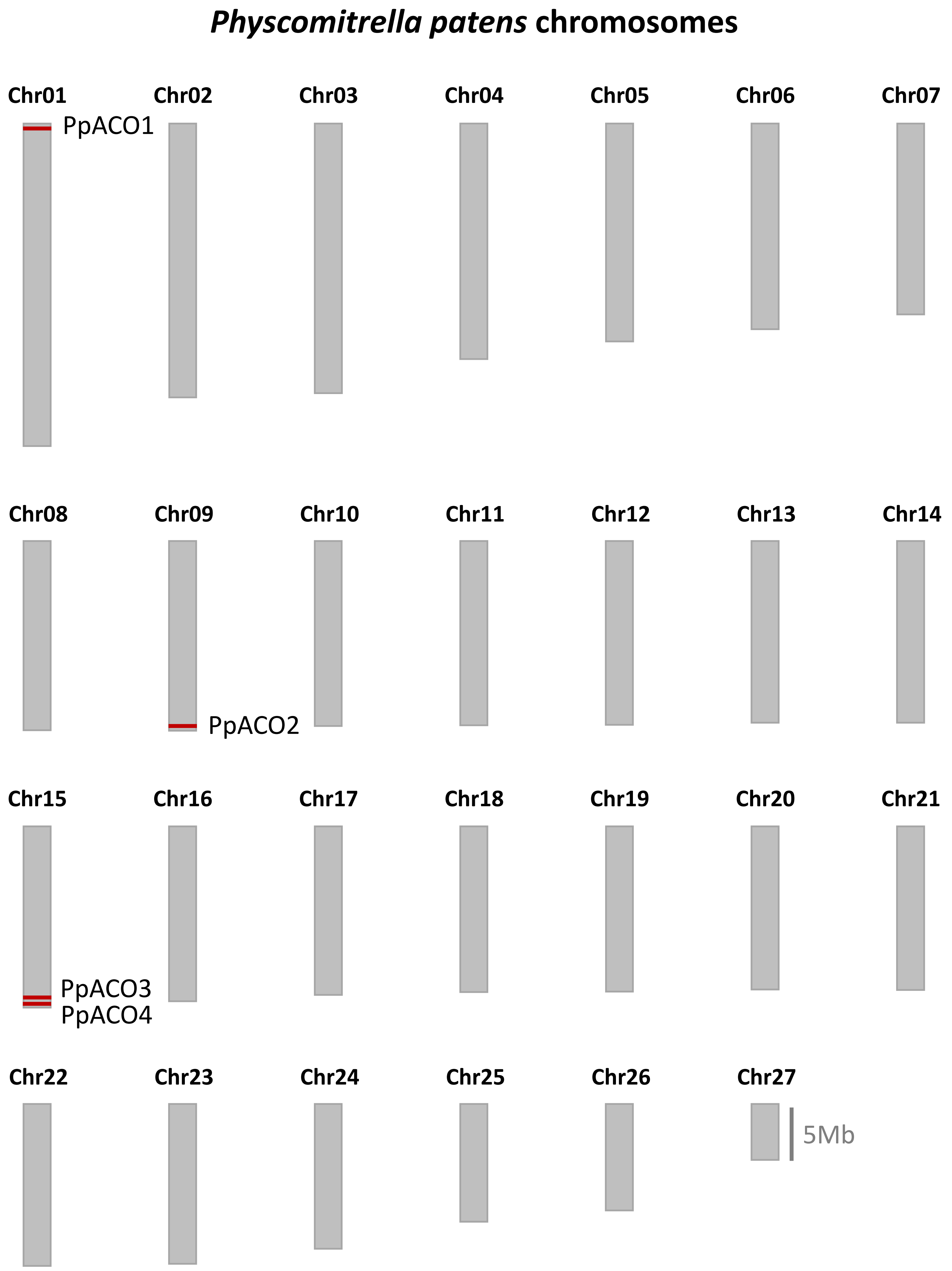

Supplement: Supplementary file 2 [file Image2.PDF]
