# Supplementary figures and images for: Molecular Evolution and Expression Divergence of the Aconitase (ACO) Gene Family in Land Plants
Source: Front Plant Sci. 2016 Dec 12;7:1879. doi: 10.3389/fpls.2016.01879 (PMC5149538; doi:10.3389/fpls.2016.01879)

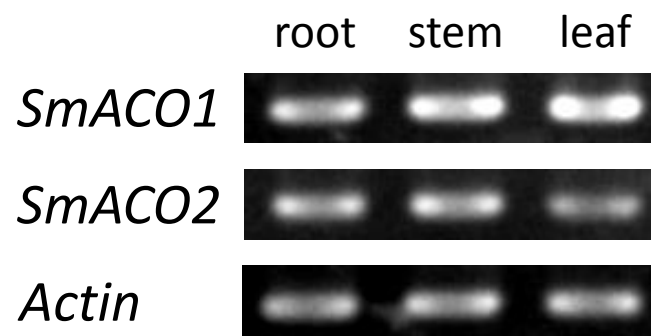

**Supplemental Figure S3. Expression of the ACO genes from different tissues of *S. moellendorffii*.**

Supplement: Supplementary file 3 [file Image3.PDF]
